# Cadherin-13 is a critical regulator of GABAergic modulation in human stem-cell-derived neuronal networks

**DOI:** 10.1038/s41380-021-01117-x

**Published:** 2021-05-10

**Authors:** Britt Mossink, Jon-Ruben van Rhijn, Shan Wang, Katrin Linda, Maria R. Vitale, Johanna E. M. Zöller, Eline J. H. van Hugte, Jitske Bak, Anouk H. A. Verboven, Martijn Selten, Moritz Negwer, Brooke L. Latour, Ilse van der Werf, Jason M. Keller, Teun M. Klein Gunnewiek, Chantal Schoenmaker, Astrid Oudakker, Alessia Anania, Sophie Jansen, Klaus-Peter Lesch, Monica Frega, Hans van Bokhoven, Dirk Schubert, Nael Nadif Kasri

**Affiliations:** 1 Department of Human Genetics, Radboudumc, Donders Institute for Brain, Cognition, and Behavior, Nijmegen, The Netherlands; 2 Department of Cognitive Neuroscience, Radboudumc, Donders Institute for Brain, Cognition and Behavior, Nijmegen, The Netherlands; 3 Division of Molecular Psychiatry, Center of Mental Health, University Hospital Würzburg, Würzburg, Germany; 4 Laboratory of Psychiatric Neurobiology, Institute of Molecular Medicine, I.M Sechenov First Moscow State Medical University, Moscow, Russia; 5 Department of Psychiatry and Neuropsychology, School for Mental Health and Neuroscience (MHeNs), Maastricht University, Maastricht, The Netherlands; 6 Department of Epileptology, ACE Kempenhaeghe, Heeze, The Netherlands; 7 Centre for Molecular and Biomolecular Informatics, Radboudumc, Radboud Institute for Molecular Life Sciences, Nijmegen, The Netherlands; 8 Centre for Developmental Neurobiology, Institute of Psychiatry, Psychology and Neuroscience, King’s College London, London, UK; 9 Department of Anatomy, Radboudumc, Donders Institute for Brain, Cognition, and Behavior, Nijmegen, The Netherlands; 10 Department of Clinical Neurophysiology, University of Twente, Enschede, The Netherlands

**Keywords:** Neuroscience, Stem cells, Cell biology

## Abstract

Activity in the healthy brain relies on a concerted interplay of excitation (E) and inhibition (I) via balanced synaptic communication between glutamatergic and GABAergic neurons. A growing number of studies imply that disruption of this E/I balance is a commonality in many brain disorders; however, obtaining mechanistic insight into these disruptions, with translational value for the patient, has typically been hampered by methodological limitations. *Cadherin-13* (*CDH13*) has been associated with autism and attention-deficit/hyperactivity disorder. CDH13 localizes at inhibitory presynapses, specifically of parvalbumin (PV) and somatostatin (SST) expressing GABAergic neurons. However, the mechanism by which CDH13 regulates the function of inhibitory synapses in human neurons remains unknown. Starting from human-induced pluripotent stem cells, we established a robust method to generate a homogenous population of SST and MEF2C (PV-precursor marker protein) expressing GABAergic neurons (iGABA) in vitro, and co-cultured these with glutamatergic neurons at defined E/I ratios on micro-electrode arrays. We identified functional network parameters that are most reliably affected by GABAergic modulation as such, and through alterations of E/I balance by reduced expression of CDH13 in iGABAs. We found that CDH13 deficiency in iGABAs decreased E/I balance by means of increased inhibition. Moreover, CDH13 interacts with Integrin-β1 and Integrin-β3, which play opposite roles in the regulation of inhibitory synaptic strength via this interaction. Taken together, this model allows for standardized investigation of the E/I balance in a human neuronal background and can be deployed to dissect the cell-type-specific contribution of disease genes to the E/I balance.

## Introduction

Neuronal network activity is controlled by a tightly regulated interplay between excitation (E) and inhibition (I). In the healthy brain, this interplay maintains a certain E/I ratio via balanced synaptic communication between glutamatergic and GABAergic neurons [1, 2], resulting in the so called “E/I balance.” A growing number of studies imply that the E/I balance is disrupted in many neurodevelopmental disorders (NDDs) [3, 4], including monogenic disorders, where the causative mutations are typically related to altered neuronal excitability and/or synaptic communication [5–7], as well as polygenic disorders, such as autism spectrum disorders (ASD) and attention-deficit hyperactivity disorder (ADHD) [4, 8]. Copy number and common variants in *Cadherin-13* (*CDH13*, also known as T-Cadherin) [9] have been associated with ASD [10], ADHD [11–14], and comorbid disorders such as depression [15] and alcohol dependence [16, 17]. CDH13 is an atypical member of the cadherin superfamily since it lacks a transmembrane and intracellular domain, and in contrast to other Cadherins, is attached to the membrane via a glycosylphosphatidylinositol (GPI) anchor [18, 19]. Because of this relatively weak connection to the outer membrane [18], CDH13 has been proposed to function as a regulatory protein, rather than an adhesion molecule [20]. Indeed, CDH13 was shown to have a role in axon guidance and outgrowth [21, 22] as well as in regulation of apoptosis during cortical development [23]. CDH13 is expressed in different cell types, dependent on brain regions, including glutamatergic, GABAergic, and serotonergic neurons [21, 24–26]. We recently showed that in the hippocampus, CDH13 is located to the presynaptic compartment of inhibitory GABAergic neurons, specifically of parvalbumin (PV^+^) and somatostatin (SST^+^) expressing neurons, and that *Cdh13* knockout (KO) mice (*Cdh13*^−/−^) show an increased inhibitory, but not excitatory synaptic input onto hippocampal CA1 pyramidal neurons [9]. In addition, these mice display deficits in learning and memory [9]. However, the mechanism via which CDH13 regulates GABAergic synapses remains unknown.

The E/I balance is particularly vulnerable to altered function and communication of GABAergic inhibitory neurons, whereas altered glutamatergic excitatory neuronal function often results in compensatory mechanisms that reinstate the E/I balance on the network level [1]. Moreover, specific classes of GABAergic neurons, such as SST^+^ and PV^+^ neurons have been found to have a particularly strong influence on the E/I balance [27, 28]. Although recent advances allowed the differentiation of human-induced pluripotent stem cells (hiPSCs) into GABAergic neurons [29–31], protocols that enable the generation of dendrite targeting SST^+^ and soma targeting PV^+^ human neurons are still challenging due to the long functional maturation of these cells [32]. Investigating E/I balance in human in vitro models for brain disorders ideally requires a model system that consists of (a) neuronal networks with a known and reproducible composition of relevant functional GABAergic and glutamatergic neuron classes, (b) GABAergic signaling that matures to the functional state of shaping network behavior by postsynaptic inhibition of neuronal activity, (c) a neuronal network that allows controlling the ratio of glutamatergic and GABAergic neurons as well as cell-type-specific manipulations of either cell-type, and (d) the possibility to assess and manipulate the neuronal communication on single neuron as well as the larger scale neuronal network level.

In this study, we investigated the role of CDH13 in maintaining E/I balance in a human neuronal model. We describe a protocol that uses direct differentiation of hiPSCs into pure populations of either induced GABAergic or induced glutamatergic neurons [33] through transcription factor-based reprogramming [30, 33, 34]. The induced GABAergic neurons included SST^+^ neurons as well as neurons expressing the PV-precursor marker protein MEF2C. When co-culturing these neurons with glutamatergic neurons over the course of 7 weeks, they exerted inhibitory modulation of postsynaptic neurons, both on a single-cell and neuronal network level. We found that reducing *CDH13* expression specifically in human GABAergic neurons increases their inhibitory control onto human glutamatergic neurons. We further show that CDH13 functionally interacts with both Integrin β1 (ITGβ1) and Integrin β3 (ITGβ3) at GABAergic synapses.

## Results

### Generation and characterization of human GABAergic neuron subtypes

**Fig. 1:**
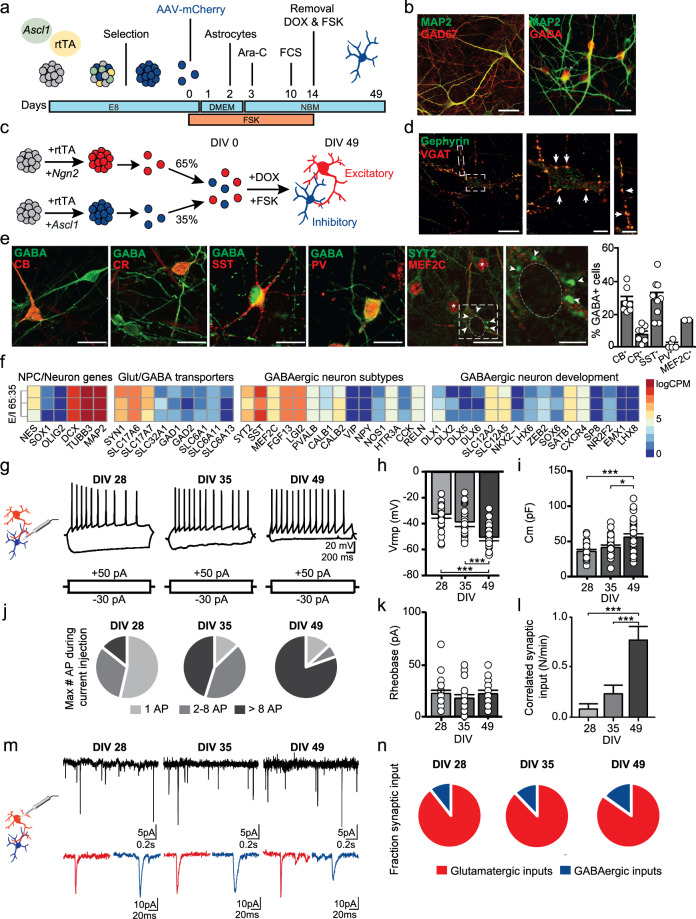
Rapid generation of human GABAergic neurons by overexpression of *Ascl1* and forskolin. **a** Culturing paradigm for the generation of induced GABAergic neurons (iGABA_A-FSK_). **b** iGABA_A-FSK_ neuron immunostaining at DIV 49 for neuronal marker MAP2 colabeled with Glutamate decarboxylase (GAD) 67 or GABA. **c** iGABA_A-FSK_ are co-cultured from DIV 0 on with iGLU_Ngn2_ to promote functional maturation (named E/I networks), in a ratio of E/I 65:35. **d** VGAT and Gephyrin co-localization in E/I networks at DIV 49. **e** Immunostaining for GABA colabeled with either calbindin (CB), calretinin (CR), somatostatin (SST), parvalbumin (PV), MEF2C (asterix), or synaptotagmin-2 (SYT2, arrowheads) in E/I networks (quantification sample size *n* = 7–9 coverslips per condition). **f** Heatmap showing expression of glutamatergic/GABAergic transporters and subtypes genes, and expression of genes important in GABAergic neuron development in E/I 65:35 networks at DIV 49 (three biological replicates from one neuronal preparation). Data represent the log-transformed counts per million (logCPM). **g** Representative firing patterns of iGABA_A-FSK_ neurons at DIV 28, 35 and 49. Analysis of iGABA_A-FSK_ membrane properties including **h** resting membrane potential (Vrmp) and **i** membrane capacitance (Cm). Analysis of action potentials evoked by step depolarization of iGABA_A-FSK_ membranes including **j** fractions of maximum number of action potentials, and **k** Rheobase. **l** Quantifications of correlated synaptic input (number of synaptic burst/minute). **m** Spontaneous glutamatergic (red inset) and GABAergic (blue inset) postsynaptic inputs (sPSCs) received by iGLU_Ngn2_. **n** Quantification of synaptic input types (DIV 28 *n* = 39, DIV 35 *n* = 38, DIV 49 *n* = 41 cells from three batches). All data represent means ± SEM. *p < 0.05; ***p < 0.001 (One-way ANOVA with Tukey correction was used to compare between DIVs). Scale bar is 20 µM, scale bars of zoom-in pictures are 6 µM.

We first developed a protocol for reproducibly generating and characterizing hiPSC-derived induced GABAergic neurons that can be co-cultured with induced glutamatergic neurons at predefined ratios. Specifically, we focused on generating SST^+^- and PV^+^-positive GABAergic neurons as CDH13 is highly expressed in these GABAergic subtypes. Moreover, these GABAergic subtypes are critical in the regulation of the E/I balance and have been implicated in NDDs [27, 28, 35]. By combining overexpression of *Ascl1* [30] in hiPSCs paired with forskolin [34, 36] (FSK, 10 μM) induction, we reliably generated GABAergic neurons (iGABA_A-FSK_, Fig. 1a, and Supplementary Fig. 1a–c) from five individual control lines that all expressed the GABAergic neuronal markers glutamic acid decarboxylase 67 (GAD67) and γ-aminobutyric acid (GABA) at days in vitro (DIV) 49 (Fig. 1b). When co-culturing iGABA_A-FSK_ neurons with iGLU_Ngn2_ neurons [33] (Fig. 1c), we identified an enrichment for SST (30%), calbindin (CB, 28%), and the PV-precursor marker protein MEF2C [37] (17%) expressing iGABA_A-FSK_ neurons (Fig. 1e). In addition, we found Synaptotagmin-2 (SYT2)-positive puncta targeting the soma of glutamatergic neurons, typically associated with synapses of PV-expressing GABAergic neurons (Fig. 1e, inset) [38]. Co-localization of the presynaptic vesicular GABA transporter (VGAT) and the postsynaptic scaffolding protein gephyrin indicated that inhibitory synapses are being formed on both the soma and dendrites (Fig. 1d). RNAseq analysis at DIV 49 further confirmed that E/I networks highly express *SST*, *MEF2C*, and genes expressed in mature fast-spiking neurons (*FGF13* [39], *LGL2* [39], *PVALB*), as well as genes coding for Glutamate and GABA transporters (*SLC17A6/7*, *GAD1/2*) and GABAergic neuron development (*DLX1-6, LHX6, ZEB2, SOX6*, Fig. 1f, and Supplementary Table 1). In summary, the generated population of GABAergic neurons is enriched for SST^+^ neurons as well as for neurons that represent the hallmarks of precursors for PV-expressing GABAergic neurons (i.e., MEF2C^+^, SYT2^+^ soma targeting synapses).

Next, we functionally characterized the maturation of these composite E/I networks at DIV 28, 35, and 49. We visually identified iGABA_A-FSK_ neurons using mCherry labeling in single-cell patch-clamp recordings (Fig. 1g–l, Supplementary Fig. 2a–f, and Supplementary Table 2). At DIV 28 and later, all recorded iGABA_A-FSK_ neurons could reliably elicit action potentials (Fig. 1g, j). As expected, during development we observed a hyperpolarization of the resting membrane potential (*V*_rmp_, Fig. 1h), as well as an increase in membrane capacitance, indicating cell growth and maturation (Fig. 1i). The rheobase remained unchanged (Fig. 1k). No effect on the level of intrinsic properties was measured in iGLU_Ngn2_ neurons cultured in the presence of iGABA_A-FSK_ neurons in E/I networks (Supplementary Fig. 2g–q and Supplementary Table 2).

In order to confirm that iGABA_A-FSK_ and iGLU_Ngn2_ functionally form an integrated network, we measured spontaneous GABAergic and glutamatergic synaptic inputs onto iGLU_Ngn2_ neurons (Fig. 1l–n). By using decay time as a threshold to separate glutamatergic and GABAergic events [30] (see “Methods,” Supplementary Fig. 2r–u and Supplementary Table 2), we show that iGLU_Ngn2_ neurons received both spontaneous glutamatergic and GABAergic synaptic inputs (spontaneous postsynaptic currents, sPSC) throughout development when recorded at a membrane potential of −60 mV (i.e., at DIV 28, 35, and 49, Fig. 1m). As a whole, during development we found a slight increase in the relative contribution of GABAergic inputs to all sPSCs (Fig. 1n) and a significant increase in the number of spontaneous synchronized synaptic inputs (bursts) onto the iGLU_Ngn2_ neurons (Fig. 1l), indicating robust integration of iGABA_A-FSK_ neurons into the E/I network as well as network-wide increased synaptic connectivity in the E/I networks over time [40].

### Functional maturation of GABAergic synaptic responses in iGLU_Ngn2_ neurons

A hyperpolarizing shift in the chloride gradient-dependent GABA reversal potential is key for enabling GABAergic synaptic inputs to modulate network activity by either shunting or hyperpolarizing inhibition and thus for establishing E/I balance during network development [41]. Local application of GABA onto iGLU_Ngn2_ somata during development revealed a prominent hyperpolarizing shift in the GABA reversal potential between DIV 35 and DIV 49 (Supplementary Fig. 3a–c). This hyperpolarizing shift of the GABA reversal potential has been shown in literature to be mediated through a decreased NKCC1:KCC2 chloride cotransporter expression ratio [41]. In accordance, in our E/I networks, the NKCC1:KCC2 expression ratio decreased between DIV 35 and 49 (Supplementary Fig. 3d–f and Supplementary Table 3). Taken together, overexpression of *Ascl1* together with FSK supplementation leads to iGABA_A-FSK_ neuron induction enriched for SST^+^, CB^+^, and PV-precursor cell types, which by DIV 49 can exert a hyperpolarizing influence on iGLU_Ngn2_ neurons.

### iGABA_A-FSK_ show inhibitory control in E/I networks recorded by micro-electrode arrays

**Fig. 2:**
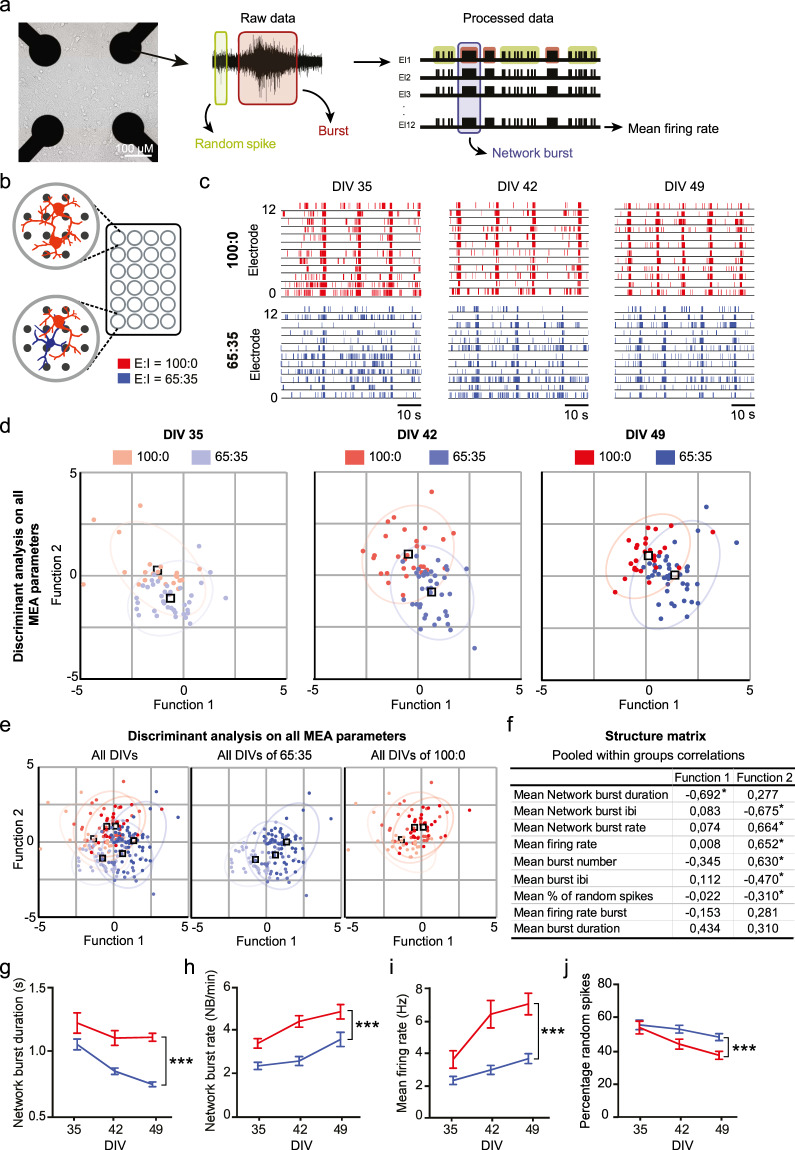
Discriminant analysis of E/I 100:0 and 65:35 networks reveal MEA parameters that reliably change depending on the hyperpolarizing GABA shift. **a** Representative image of E/I network density on micro-electrode arrays (MEAs). Schematic representation of spontaneous electric activity patterns measured on MEAs. **b** iGLU_Ngn2_ alone (E/I ratio: 100:0) or in co-culture with iGABA_A-FSK_ (E:I ratio: 65:35) were recorded side by side on a multiwell MEA. **c** Representative raster plots showing 60 s of activity from 100:0 (red) or 65:35 (dark blue) cultures at DIV 35, 42, and 49. Canonical scores plots based on discriminant analyses of all nine analyzed MEA parameters (methods) for E/I 100:0 and 65:35 networks **d** at all DIVs separate, **e** all DIVs combined (first panel) only E:I 65:35 cultures at all DIVs (second panel) and only E:I 100:0 cultures at all DIVs (third panel). **f** Structure matrix values showing which parameters explain the changes in neuronal network activity. Significantly changed parameters are marked with an Asterix. Quantifications of neuronal network activity including **g** network burst duration, **h** network burst rate, **i** mean firing rate, and **j** percentage of random spikes (E:I 100:0 DIV 35 *n* = 25, DIV 42 *n* = 30, DIV 49 *n* = 29; 65:35 DIV 35 *n* = 40, DIV 42 *n* = 39, and DIV 49 *n* = 38 individual wells from six individual neuronal preparations). DIV days in vitro. All data represent means ± SEM. ***p < 0.001 (mixed model Two-way ANOVA was performed between DIVs, *p* values were corrected for multiple comparisons using Sidak’s). IBI inter-burst interval.

Having established a protocol for generating iGABA_A-FSK_ neurons that can exert a hyperpolarizing (inhibitory) influence on iGLU_Ngn2_ neurons, we next investigated how these GABAergic neurons functionally modulate neuronal network development. We performed a comprehensive network analysis comparing two different network compositions of either iGLU_Ngn2_ alone (E/I ratio: 100:0), or in co-culture with iGABA_A-FSK_ neurons (E/I ratio: 65:35) on multielectrode arrays (MEAs). Neuronal networks recorded on MEAs can display three distinctive patterns of activity, namely (i) random spiking activity (Fig. 2a, green box), (ii) activity that is organized into a local burst (i.e., high frequency trains of spikes, Fig. 2a, red box), and (iii) network-wide bursting (i.e., bursts detected in all channels, Fig. 2a, purple box) during development. First, we confirmed that at DIV 49 treatment of E/I networks with 100 µM GABA completely abolished neuronal network activity (Supplementary Fig. 4a, b). Next, we compared the MEA recordings between the two network compositions side by side at DIV 35, 42, and 49 (Fig. 2b, c). Using discriminant analysis of nine independent MEA parameters at all time-points, we identified network burst duration (NBD), followed by network burst rate (NBR), mean firing rate (MFR), and the percentage of random spikes (PRS) as the main parameters that explain the significant differences in network activity between E/I 100:0 and E/I 65:35 networks (Fig. 2d–f). Specifically, over development (i.e. at DIV 35, 42 and 49) we detected a shortening of the NBD (Fig. 2g), as well as a reduced NBR (Fig. 2h) and MFR (Fig. 2i), in contrast to an increased PRS (Fig. 2j) in E/I 65:35 networks as compared to E/I 100:0 networks. Interestingly, all of these network activity parameters only became significantly different between E/I 65:35 and E/I 100:0 after DIV 42 (Supplementary Table 4). The time-point for these differences to become significant indicates that the hyperpolarizing shift of the GABA reversal potential and thereby the maturation of the inhibitory system is underlying the different trajectories in functional network development between E/I 65:35 and E/I 100:0 networks. Importantly, we show that this change in network activity is reproducible amongst E/I networks generated with five independent *Ascl1*-transduced healthy control hiPSCs (Supplementary Fig. 1d–h and Supplementary Table 5). Together, our results show that we can monitor and quantify the modulation of network activity by mature iGABA_A-FSK_ neurons during development on MEAs using a well-defined set of MEA parameters.

### iGABA_A-FSK_ exhibit scalable inhibitory control onto the neuronal network

**Fig. 3:**
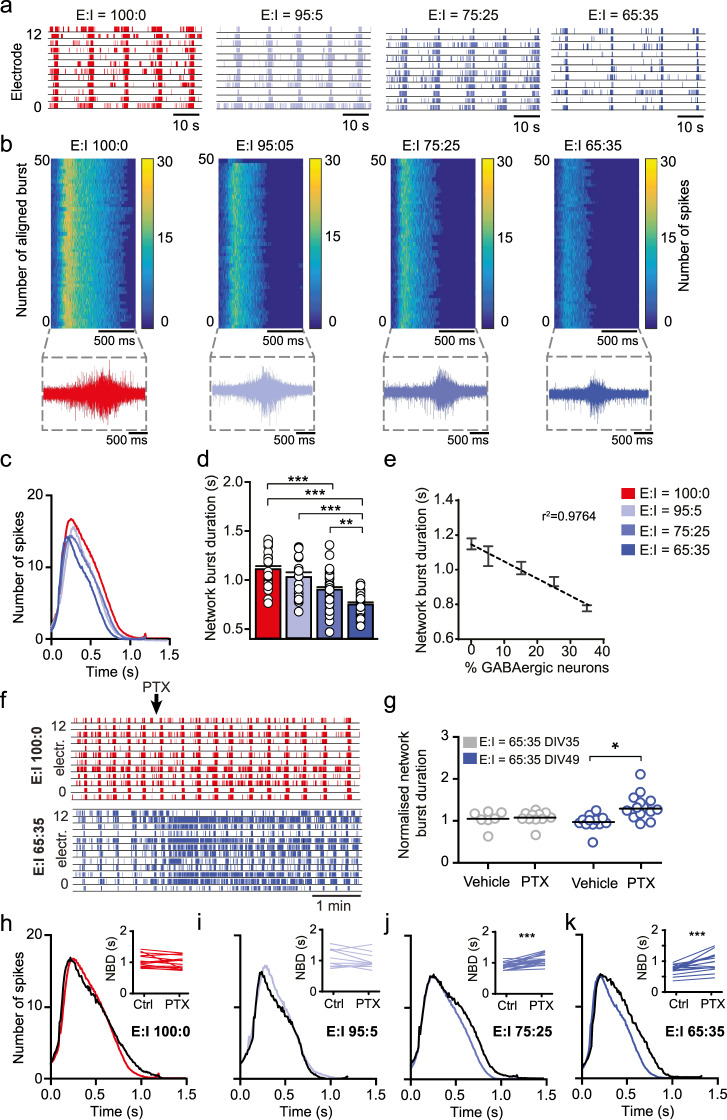
iGABA_A-FSK_ show scalable functional inhibition on the neural network at DIV 49. **a** Representative raster plots showing 60 s of activity recorded from E:I 100:0 (red), 95:5 (light blue), 75:25 (blue), or 65:35 (dark blue) networks at DIV 49. **b** Representative network burst alignment from one recording of E:I 100:0, 95:5, 75:25, or 65:35 networks, color code represents the number of spikes. Inset: representative network burst. **c** Average network burst shape of representative cultures from 100:0, 95:5, 75:25, or 65:35 networks at DIV 49 (E:I 100:0 cultures *n* = 20, 95:5 *n* = 12, 75:25 *n* = 23, and 65:35 *n* = 26 individual wells. For E:I 65:35 networks *p* = 0.008, multiple *t*-test on bins using Holm–Sidak method). **d** Quantification of the average network burst duration of E:I 100:0, 95:5, 75:25, and 65:35 networks (E:I 100:0 *n* = 29, 95:5 *n* = 20, 75:25 *n* = 38, and 65:35 *n* = 38 individual wells, Kruskal–Wallis Two-way ANOVA was performed between ratios at DIV 49, corrected using the Dunn’s method). **e** Linear regression plot of the average network burst duration from 100:0, 95:5, 85:15, 75:25, or 65:35 cultures at DIV 49 (*y* = −9.628*x* + 1109, *p* = 0.0119). **f** Representative raster plots of 5 min showing the effect of acute 100 µM picrotoxin (PTX) treatment on E/I 100:0 and 65:35 networks at DIV 49. **g** Normalized network burst duration of E/I 65:35 networks treated acutely with vehicle or PTX at DIV 35 and 49, normalized to their respective baseline recording (DIV 35 + vehicle *n* = 8; DIV 35 + PTX *n* = 11; DIV 49 + vehicle *n* = 12, and DIV 49 + PTX *n* = 15 individual wells, Mann–Whitney test with post hoc Bonferroni correction was performed). Quantification of network burst shape after acute PTX treatment in **h** 100:0, **i** 95:5, **j** 75:25, and **k** 65:35 cultures at DIV 49 (black line indicates the average burst shape of wells treated with PTX, E:I 100:0 *n* = 9, 95:5 *n* = 6, 75:25 *n* = 12, and 65:35 *n* = 11 individual wells, 100:0 *p* = 0.5582, 95:5 *p* = 0.1857, 75:25 *p* = 0.1050, and 65:35 *p* = 0.0013, multiple *t*-test on bins using the Holm–Sidak method). Inset: paired *t*-test of the mean network burst duration before and after treatment with PTX (E:I 100:0 cultures *n* = 15, 95:5 *n* = 10, 75:25 *n* = 19, and 65:35 *n* = 15 individual wells). DIV days in vitro. All data represent means ± SEM. *p < 0.05, *p < 0.01, ***p < 0.001.

We evaluated to which extent the inhibition-mediated changes on the aforementioned MEA parameters depends on the specific ratio of iGLU_Ngn2_:iGABA_A__-FSK_ present in our neuronal networks. To this end we co-cultured four different E/I ratios: 100:0, 95:5, 75:25, and 65:35 (Supplementary Fig. 1c) on MEAs and recorded spontaneous activity at DIV 49. In all conditions, the number of iGLU_Ngn2_ neurons was kept constant, whilst the number of iGABA_A-FSK_ was changed. Our data show that the length of NBD was negatively correlated to the percentage of iGABA_A-FSK_ neurons (Fig. 3a–e). In addition, to the shortening of the NBD with increasing percentages of iGABA_A-FSK_ in the networks, we also detected network bursts to be composed of fewer detected spikes (Fig. 3b, c). Furthermore, increasing percentages of iGABA_A-FSK_ in the networks led to a significant reduction in the MFR (Supplementary Fig. 4c) and NBR (Supplementary Fig. 4e) as well as an increase in PRS (Supplementary Fig. 4d and Supplementary Table 6).

We showed that iGABA_A-FSK_ neurons shape network burst activity at DIV 49 through inhibition. We next investigated how acute removal of inhibitory control alters the NBD compared to networks that lacked inhibitory control during development (i.e., iGLU_Ngn2_ only cultures). Following acute treatment with either vehicle or 100 µM Picrotoxin (PTX), the NBD did not change in the 100:0 E/I networks at DIV 49 (Fig. 3f, top panel). In contrast, PTX significantly increased the NBD and MFR of 65:35 E/I networks (Fig. 3f, bottom panel). In accordance with our data on the hyperpolarizing shift of the GABA reversal potential during development, in these 65:35 E/I networks acute treatment with PTX at DIV 35 did neither affect the NBD (Fig. 3g) nor the MFR (Supplementary Fig. 4f). Moreover, the impact of PTX treatment on the NBD and MFR was again scalable to the ratio of iGABA_A-FSK_ neurons present (Fig. 3h–k and Supplementary Fig. 4h). We identified a similar significant increase of the MFR and NBD with an additional increase of the NBR in E/I networks exposed to 40 µM Bicuculline (BIC) at DIV 70 (Supplementary Fig. 4g and Supplementary Table 7). Finally, we infected E/I networks with an AAV expressing Channelrhodopsin-2 in either iGLU_Ngn2_ or iGABA_A-FSK_ neurons. Optogenetic activation of iGLU_Ngn2_ neurons at DIV 49 resulted in an increase in MFR (Supplementary Fig. 4i), whereas optogenetic activation of iGABA_A-FSK_ neurons reduced the MFR (Supplementary Fig. 4j and Supplementary Table 7). Together, these data show that iGABA_A-FSK_ neurons at the network level exert robust inhibitory control at DIV 49.

### Knockdown of *CDH13* increases inhibitory control onto neuronal networks

**Fig. 4:**
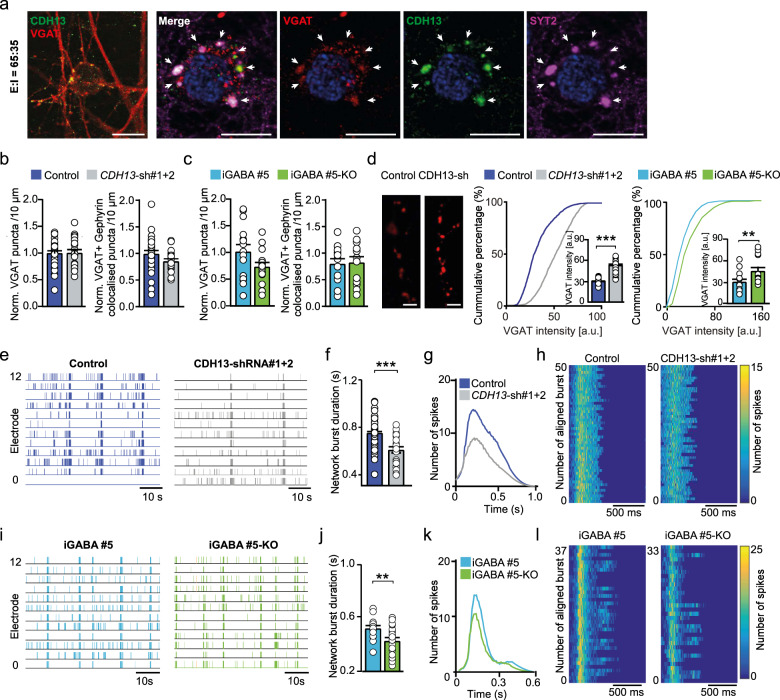
Knockdown of CDH13 in iGABA_A-FSK_ leads to increased inhibition in E/I networks. **a** Co-labeling of VGAT (red), CDH13 (green), and SYT2 (purple) in E/I 65:35 controls at the inhibitory presynapse (scale bar 10 µM). Normalized number of total VGAT-positive puncta (left) and normalized number of VGAT and Gephyrin co-localized puncta (right) in **b** *CDH13*-sh#1 + 2-transduced networks or **c** iGABA#5 and iGABA#5-KO networks at DIV 49. **d** Representative VGAT staining in E/I 65:35 control and CDH13-deficient networks at DIV 49 (scale bar 6 µM) and quantification of VGAT puncta intensity (arbitrary units, control *n* = 24, *CDH13*-sh#1 + 2 *n* = 26, iGABA#5 *n* = 13, and iGABA#5-KO *n* = 15 images from three individual neuronal preparations. Mann–Whitney test with post hoc Bonferroni correction was performed). **e, i** Representative raster plots showing 60 s of electrophysiological activity recorded from E/I 65:35 control and CDH13-deficient cultures at DIV 49. **f, j** Quantification of the average network burst duration in E/I 65:35 control and CDH13-deficient networks (Control *n* = 49, *CDH13*-sh#1 + 2 *n* = 31, iGABA#5 *n* = 20, and iGABA#5-KO *n* = 21 individual wells from three neuronal preparations. Mann–Whitney test with Bonferroni correction was performed). **g, k** Average network burst shape of representative cultures from E/I 65:35 control and CDH13-deficient networks at DIV 49 (Control *n* = 26 and *CDH13*-sh#1 + 2 *n* = 12 individual wells, *p* = 0.00071; iGABA#5 *n* = 19 and iGABA#5-KO *n* = 20 individual wells, *p* = 0.235. Multiple *t*-test on bins were performed using the Holm–Sidak method). **h, l** Representative network burst alignment from one recording of E/I 65:35 control and CDH13-deficient networks at DIV 49, color code represents # spikes. All data represent means ± SEM. *p < 0.05; **p < 0.01; ***p < 0.001. DIV days in vitro.

To investigate the role of *CDH13* in maintaining E/I balance in human neurons, we first verified its expression in 65:35 E/I networks. Amongst many other disorder-related genes with a prominent influence on the E/I balance such as Neuroligin (*NLGN*) and Neurexin (*NRXN*) [42], we also found *CDH13* to be expressed in these E/I networks (Supplementary Fig. 5a). Moreover, we found CDH13 to be co-localized with VGAT and SYT2 (Fig. 4a), demonstrating that, as in rodent neurons [9], also in human iPSC-derived E/I networks CDH13 is localized to inhibitory presynapses. Of note, iGLU_Ngn2_-only networks did not express CDH13, confirming that CDH13 is exclusively expressed in iGABA_A-FSK_ neurons (Supplementary Fig. 5b).

After confirming CDH13 expression in control 65:35 E/I networks, we investigated the functional consequences of reduced CDH13 expression in GABAergic neurons. To this end we employed two independent validated short hairpin RNAs (shRNA) to downregulate *CDH13* expression [26] specifically in iGABA_A-FSK_ neurons, by only infecting *Ascl1*-expressing hiPSCs prior to co-culturing (Supplementary Fig. 5c and Supplementary Tables 8, 9). In addition, we used an *CDH13* KO hiPSC line previously generated with CRISPR/Cas9 genome editing [43] and differentiated these into GABAergic neurons (iGABA#5-KO). These iGABA#5-KO GABAergic neurons were co-cultured with glutamatergic neurons derived from its isogenic control line (iGLU#3) to study loss of CDH13 only in GABAergic neurons (from here on only referred to as iGABA#5-KO). We confirmed that an equal amount of GABAergic neurons was generated in iGABA#5 (Control E/I networks) and iGABA#5-KO networks (iGABA#5 = 27.7 ± 0.99%, iGABA#5-KO = 28.5 ± 1.82%). We next assessed if reduced CDH13 expression in GABAergic neurons caused an altered formation of GABAergic synapses. At DIV 49, CDH13-deficient networks showed neither changes in the number of inhibitory presynapses identified by VGAT labeling, nor in inhibitory synapses identified by juxtaposed VGAT/Gephyrin puncta as compared to control networks (Fig. 4b, c). However, CDH13-deficient networks showed a striking increase in the mean intensity of VGAT puncta (Fig. 4d), suggesting that loss of CDH13 does not affect the synapse density but rather results in increased inhibitory synaptic strength.

As neural network activity of E/I networks is scalable to the level of GABAergic modulation, we next assessed the impact of CDH13 deficiency in iGABA_A-FSK_ neurons on the level of network activity at DIV 49. Lentiviral infection as such did not affect network activity of control E/I networks (non-treated vs. empty vector, Supplementary Fig. 5d). However, networks transduced with two shRNAs (*CDH13*-sh#1 + 2) showed a significantly reduced NBD together with an altered average burst shape and less detected spikes within a network burst (Fig. 4e–h). Similar alterations in the NBD and network burst shape were found in networks transduced with only one of the shRNAs (i.e., sh#1 or sh#2, Supplementary Fig. 5e–g). Furthermore, the *CDH13*-sh#1 + 2-transduced networks showed a significantly reduced NBR, while the PRS was significantly increased (Supplementary Table 10).

To confirm that these changes in network burst shape are caused by loss of CDH13 in GABAergic neurons, we studied neuronal network activity between iGABA#5 and iGABA#5-KO networks at DIV 49. Similar as in shRNA-transduced networks, we detected a significantly reduced NBD together with altered average burst shape and less detected spikes within a network burst in iGABA#5-KO networks as compared to iGABA#5 networks (Fig. 4i–l). In addition, we again detected a reduced NBR between iGABA#5 and iGABA#5-KO networks, while the PRS again significantly increased (Supplementary Table 10). Taken together, the increased VGAT intensity paired with the changes in neuronal network parameters suggest an increased inhibitory drive upon the neuronal network due to loss of CDH13 in GABAergic neurons.

To confirm that loss of CDH13 results in increased GABAergic modulation, we measured GABAergic sPSCs (sIPSCs) at DIV 70 in both *CDH13*-shRNA transduced networks as well as in iGABA#5-KO E/I networks on a single-cell level. We detected an increase in sIPSC amplitude and/or sIPSC frequency in both *CDH13*-sh#1 or sh#2transduced networks as well as in iGABA#5-KO E/I networks (Supplementary Fig. 5h–j and Supplementary Table 10). These results confirm that loss of CDH13 causes increased GABAergic synaptic input, further supporting that CDH13 is a negative regulator of inhibitory synaptic function.

### CDH13 regulates inhibitory synaptic strength via interaction with ITGβ1 and ITGβ3

**Fig. 5:**
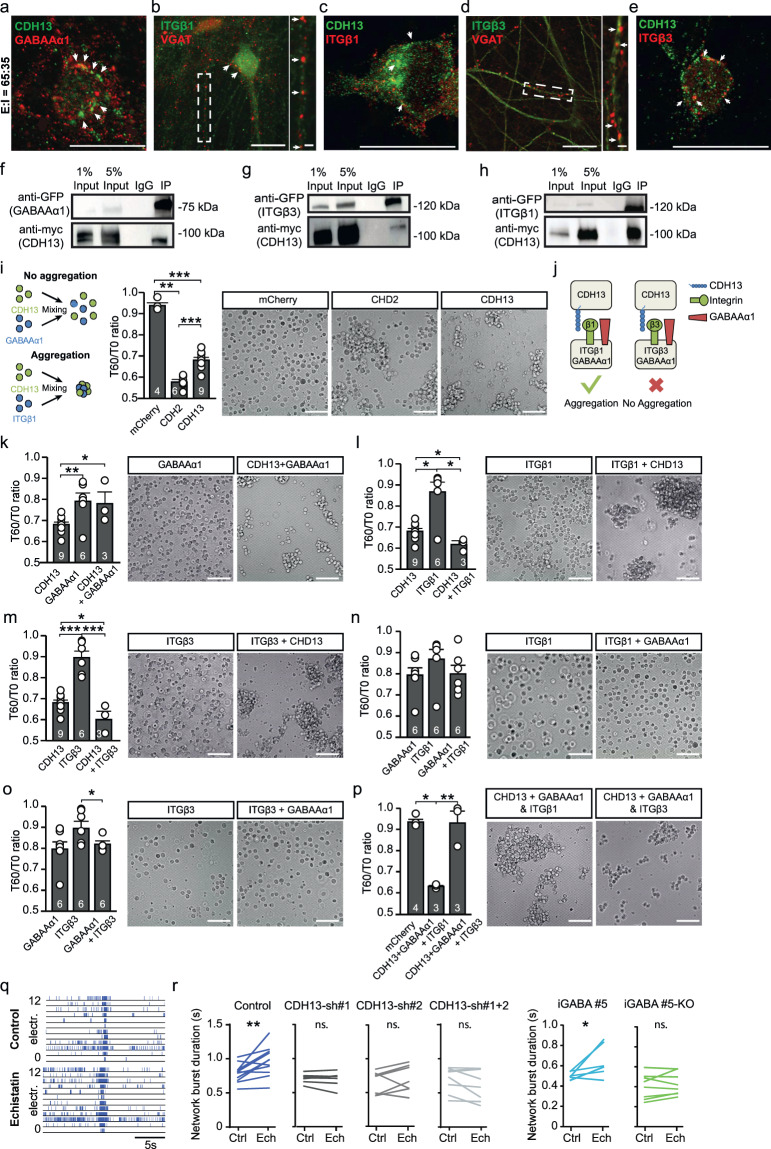
CDH13 interacts with integrin β1 (ITGβ1) and ITGβ3 in vitro. Representative co-localizations of **a** GABAAα1 with CDH13, **b** Integrin (ITG) β1 with VGAT, **c** ITGβ1 with CDH13, **d** ITGβ3 with VGAT, and **e** ITGβ3 with CDH13 in E:I 65:35 networks (scale bar 20 µM, zoom-in pictures 6 µM). Western blot showing co-immunoprecipitation of **f** CDH13 with GABAAα1, **g** CDH13 with ITGβ3, and **h** CDH13 with ITGβ1 in HEK cells. **i** Left Panel: Schematic representation of a cell aggregation assay with non-aggregating (top) and strongly aggregating (bottom) cells. Right Panel: Representative images and quantification of cell aggregation for indicated proteins in HEK cells (sample size in figure, scale bar 50 µM). **j** Visual summary of cell aggregation data: in the presence of GABAAα1, integrin (ITG) β1 expressing HEK293T cells, but not ITGβ3 expressing HEK293T cells, aggregate with CDH13 expressing HEK293T cells. **k**–**p** Representative images and quantification of cell aggregation for indicated proteins in HEK cells (sample size in figure, scale bar 50 µM). **q** Representative raster plots of E:I 65:35 control and CDH13-deficient networks treated with 100 µM Echistatin. **r** Quantification of the network burst duration of pre- and post-echistatin treated E:I 65:35 control and CDH13-deficient networks (Control *n* = 16, *CDH13*-sh#1 *n* = 7 wells, *CDH13*-sh#2 *n* = 7 wells, *CDH13*-sh#1 + 2 *n* = 8 wells, iGABA#5 *n* = 7 wells and iGABA#5-KO *n* = 8 individual wells from three neuronal preparations. Paired *T*-test was performed between pre- and post-echistatin treatment conditions). All data represent means ± SEM. *p < 0.05; **p < 0.01; ***p < 0.001. IP immunoprecipitation.

The observed increase of VGAT expression in CDH13-deficient networks implies that CDH13 is a negative regulator of synapse function; however, the underlying mechanism is unknown. CDH13 is a GPI-anchored protein, which suggests that binding to other membrane bound proteins is required to exert its function [18, 19]. In agreement with rodent data [9], we showed that in hiPSC-derived E/I networks, CDH13 expression is restricted to GABAergic neurons; therefore, a heterophilic interaction is likely to be required for CDH13 to exert its function. Previous co-immunoprecipitation studies in endothelial cells identified the GABAA receptor α1 subunit (GABAAα1) and ITGβ3 [44] as potential interaction partner for CDH13. Overexpression of CDH13 has also been shown to increase ITGβ1 expression in squamous carcinoma cells [45], even though a direct interaction has not been reported. Interestingly, ITGβ1 and ITGβ3 have opposite functions in regulating synaptic dwell time of glycine receptors in spinal cord neurons, bidirectionally regulating the synaptic strength of these inhibitory synapses [46]. Both, ITGβ1 and ITGβ3, are expressed in glutamatergic neurons, where they play a role in regulating glutamatergic synaptic function though the modulation of AMPARs [47, 48]. However, until now a role in the regulation of GABAergic synaptic function in glutamatergic neurons has not been described for these integrins. PV^+^ synapses are enriched for the GABA receptor subunit α1 (GABAAα1 [49]). Therefore, we hypothesized that CDH13 may play a role in regulation of GABAergic synapse stability via direct interaction with GABAAα1, ITGβ1, or ITGβ3. We first assessed the cellular localization of CDH13, ITGβ1, ITGβ3, and GABAAα1 in our E/I networks (Fig. 5a–e). Whereas CDH13 co-localized with VGAT (Fig. 4a) in the presynaptic terminal, GABAAα1 localized juxtapose of CDH13 (Fig. 5a). ITGβ1 (Fig. 5b, c) and ITGβ3 (Fig. 5d, e) localized juxtapose of VGAT and CDH13, suggestive of a postsynaptic localization. Next, we confirmed the interactions between ITGβ1, ITGβ3, GABAAα1, and CDH13 by co-immunoprecipitation experiments, using lysates from a human embryonic kidney cell line (HEK293) expressing GFP-tagged GABAAα1, GFP-tagged ITGβ1 or ITGβ3, and myc-tagged CDH13 (Fig. 5f–h). Finally, we confirmed an endogenous interaction between ITGβ1 and CDH13 by co-immunoprecipitation experiments, using lysates from E/I networks at DIV 49 (Supplementary Fig. 5k).

### Differential roles for ITGβ1 and ITGβ3 in cell adhesion assays

Since our data indicate that CDH13, ITGβ3, and GABAAα1 co-localized at the same synapse, we wanted to know if these proteins are able to play a role in cell adhesion. To this end we used a cell adhesion assay [50]. In this assay, we transfected HEK293T cells with a vector expressing CDH13, ITGβ1, ITGβ3, or GABAAα1 and quantified the degree of aggregation at two different time-points and calculated the ratio (T60/T0 ratio). As negative and positive control for cell adhesion, we transfected HEK293T cells, respectively, with mCherry or Cadherin 2 (CDH2), of which the relative strengths of binding are known (Fig. 5i) [18, 51]. In line with literature, CDH2 showed a strong aggregation [51, 52] (Fig. 5i, low T60/T0: 0.58 ± 0.01), whereas mCherry-expressing HEK293T cells showed very little aggregation (Fig. 5i, high T60/T0: 0.94 ± 0.01). Homophilic interactions of CDH13 have been proposed, but are predicted to be weak compared to CDH2 homophilic interactions [18, 19]. Indeed, in our assay, CDH13-expressing HEK293T cells showed an intermediate value (Fig. 5i and Supplementary Table 11) and show that this assay has the sensitivity to distinguish between different strengths of cell adhesion.

We then investigated the interactions between CDH13, ITGβ1, ITGβ3, or GABAAα1. GABAAα1 was co-transfected with GABAAβ3 to ensure surface expression of these proteins [53]. GABAAα1/β3-expressing HEK293T cells showed a weak homophilic interaction (Fig. 5k). We then combined HEK293T cells expressing CDH13 or GABAAα1/β3; however, the resulting T60/T0 ratio indicated no heterophilic adhesion between these proteins (Fig. 5k and Supplementary Table 11). Conversely, while ITGβ1 shows no homophilic interaction, consistent with previous reports [54], CDH13 and ITGβ1 showed a heterophilic interaction (Fig. 5l and Supplementary Fig. 5l). ITGβ3 showed the same pattern as ITGβ1, displaying a stronger interaction with CDH13 (Fig. 5m and Supplementary Fig. 5l) than either CDH13 or ITGβ3 alone. We next assessed the interaction between integrins and GABAα1/β3 in transsynaptic conformation. A mix of ITGβ1 expressing and GABAα1/β3-expressing cells did not show interaction (Fig. 5n). We also found no interaction between ITGβ3 and GABAAα1/β3 expressing cells (Fig. 5o). Finally, we investigated cell aggregation using a protein arrangement as expected in vivo. We expressed either ITGβ1 or ITGβ3 together with GABAAα1/β3 in one population of HEK293T cells, representing the postsynaptic side. To represent the presynaptic side, we transfected HEK293T cells with CDH13 (Fig. 5p). Surprisingly, we found that while ITGβ1- and GABAAα1/β3-expressing cells displayed a strong interaction with CDH13-expressing cells, ITGβ3- and GABAAα1/β3-expressing cells did not interact with CDH13-expressing cells (Fig. 5p, Supplementary Fig. 5l, and Supplementary Table 11). In conclusion, while both ITGβ1 and ITGβ3 show interaction with CDH13, co-expression of GABAAα1/β3 with the integrins leads to a loss of interaction between CDH13 and ITGβ3, specifically (Fig. 5j).

If ITGβ1 and ITGβ3 play a role in inhibitory synapse stabilization via their interaction with CDH13, disruption of integrin function should affect inhibitory transmission. In order to test this on the functional level, we applied 100-nm Echistatin, an inhibitor of ITGβ1 and ITGβ3 [55], to E/I networks recorded at DIV 49 on MEA. Blocking ITGβ1/3 interaction increased NBD and MFR in control E/I networks, indicating that ITGβ1/3 play a role in maintaining inhibitory strength in control networks. Interestingly, Echistatin had no effect on CDH13-deficient networks compared to vehicle-treated cells (Fig. 5q, r, Supplementary Fig. 5m, and Supplementary Table 12). Together, these data indicate that ITGβ1/ITGβ3 play a critical role in inhibitory synapse maintenance, and that this role is dependent on the presence of CDH13.

## Discussion

In this study, we describe a human in vitro neuronal model system for investigating the function of CDH13 in the maintenance of E/I balance. In humans, copy number and common variants of *CDH13* have been identified in large datasets of ASD and ADHD patients [10, 25, 56], and in rodents has been found to alter E/I balance on the single-cell level [9]. Since the first postulation of an increased E/I ratio in ASD [57], an increasing amount of studies has shown that altered E/I balance contributes to many NDDs [2]. Interestingly, evidence from both animal models and human studies suggest that an altered function of PV^+^ GABAergic neurons is a common unifying pathway for common forms of NDDs [4, 27, 32]. Although several efforts have been made to generate PV^+^, fast-spiking GABAergic neurons from hiPSCs, their generation has been proven challenging [32]. Here we show that *Ascl1* overexpression and FSK supplementation resulted in ~30% SST^+^ GABAergic neurons. Even though a large population of distinct PV-expressing neurons was absent, 15–20% of the GABAergic neurons were expressing MEF2C, a marker for immature PV^+^ neurons [37]. Together with the existence of soma targeting SYT2-positive GABAergic synapses onto iGLU_Ngn2_ neurons in our cultures and the recent finding that in both, SST^+^ and PV^+^ GABAergic neurons synapse targeting specificity follows distinct molecular programs [39], this implies that these MEF2C^+^ neurons represent PV^+^ precursor cells [38]. PV^+^ GABAergic neurons are known to follow a maturation trajectory that is likely to exceed the developmental time window covered in most in vitro culture studies and for which the current culturing conditions may not be optimally set [58]. However, even though in comparison to mature fast-spiking PV^+^, our non-fast-spiking MEF2C expressing PV^+^ precursor cells will consequently differ in the manner of spike output, they can still provide synaptic GABAergic inputs onto postsynaptic somatic domains.

After establishing a protocol that generates a reproducible composition of GABAergic neuronal classes that can form the relevant GABAergic circuitry, we confirmed the functional maturation of GABA signaling in the E/I networks. In vivo, the emergence of functional GABAergic inhibition via GABAA receptors is facilitated by a hyperpolarizing shift in the chloride reversal potential during development mediated through activity-dependent increase in the ratio of KCC2:NKCC1 chloride cotransporter expression in neurons [59]. Multiple studies have evaluated the generation of iGABA neurons based on the expression of GABAergic markers and synaptic GABA release [29–31]. However, to our knowledge, it has not been shown before that using direct differentiation of hiPSC into composite E/I networks, iGABA_A-FSK_ develop into neurons that functionally modulate iGLU_Ngn2_ network activity by GABA-mediated postsynaptic shunting inhibition and/or hyperpolarizing inhibition. This is not only important for network phenotyping, but is also essential for iGLU_Ngn2_ maturation and the maintenance of the E/I balance [60]. Our data demonstrate that the generated E/I networks receive glutamatergic as well as GABAergic synaptic inputs and indeed show a decrease in the NKCC1:KCC2 ratio during development. At the functional level, we could correlate this with a hyperpolarizing shift of the GABA reversal potential, indicating iGABA_A-FSK_ neurons in mature in vitro E/I networks can functionally modulate network activity in E/I networks.

This leaves the question regarding how to assess E/I balance at a neuronal network level. One well-established model to generally assess neuronal network activity in vitro are cultures growing on MEAs [61–63]. Indeed, MEAs have shown to be a powerful tool to elucidate the contribution of receptors of excitatory and inhibitory synaptic transmission to spontaneous network activity in rodent in vitro cultures [64]. Here we show the development of hiPSC-derived E/I networks over time, and describe network parameters that most prominently illustrate the modulation of hyperpolarizing/shunting inhibition by iGABA_A-FSK_ neurons. In relation to the temporal aspects of the hyperpolarizing shift in the chloride-gradient-dependent GABA reversal potential, we show a decrease of the NBD, MFR, and NBR and an increase in the PRS over development from DIV 35 to 49, which are in line with previously published work in rodent and human E/I networks on MEA [61, 63]. In particular, the shortening of the NBD has been demonstrated to be a hallmark of mature GABA-mediated signaling in neuronal networks [61, 64, 65], mainly by reducing the intra burst activity, which in turn scales down the Mg^2+^ block release from the NMDAR pore [64, 66]. In our E/I cultures, we could not only reproduce the maturation-dependent effects of GABAergic signaling on network bursts, but also demonstrated that these effects are scalable to the amount of inhibitory neurons in the E/I cultures: we were able to show a direct correlation between the different network parameters and the amount of inhibition. We furthermore showed that these composition-specific changes in the NBD were reproducible amongst E/I networks composed of five independent *Ascl1*-transduced healthy control hiPSC. However, for other parameters (i.e., MFR and NBR), we did observe some line-to-line variation between these five *Ascl1*-transduced control lines. In line with our results, we have previously shown that certain MEA parameters extracted from iGLU_Ngn2_ neuronal networks only show little variation, whereas other parameters (including the MFR) are variable between control lines derived from ten individual healthy subjects [67]. These results warrant the use of multiple MEA parameters and multiple control lines while characterizing neuronal phenotypes in E/I networks on MEA. Furthermore, we advise to always first perform a basic characterization of the excitatory and inhibitory neurons to define those parameters that stably change upon the maturation of GABAergic inhibition. In addition to line-to-line variation on the level of spontaneous activity parameters, we also identified variations in the response of these E/I networks to GABA inhibitory agents such as PTX or BIC. Therefore, we advise to include several GABA inhibitory agents during the basic characterization of E/I networks before using this model as a phenotyping platform [68, 69]. Finally, several factors aside from the use of new hiPSC lines can introduce variation in the data, such as experimental design or data analysis settings. We recently published a set of guidelines to improve the variability in MEA data, which will also apply to this model (see “Methods” and ref. [67]).

Using this model, we studied the cell-type-specific contribution of CDH13 in iGABA_A-FSK_ neurons. When comparing control networks with networks in which CDH13 expression is specifically reduced in only iGABA_A-FSK_ neurons, we found several lines of evidence that show that CDH13 deficiency increased inhibitory control at the network level, which is in line with the synaptic phenotypes found in hippocampal CA1 neurons of *Cdh13*^*−/−*^ mice [9]. With keeping the scalable consequences of the amount of GABAergic neurons on network behavior in mind, both CDH13-shRNA transduced as well as iGABA#5-KO networks clearly imply an elevated impact of GABAergic signaling on the E/I cultures. One prominent feature illustrating the elevated impact of GABAergic signaling was the strong shortening of NBD, most likely mediated by elevated suppression of within burst spiking and consequently the suppression of late NMDAR-dependent phase of the bursts [66]. In addition to the shortening of the NBD on MEA, we found a clear increase of VGAT puncta intensity, as well an increased sIPSC amplitude and/or frequency in these CDH13-deficient networks, supporting the evidence that CDH13 is a negative regulator of inhibitory synaptic function.

At the molecular level, we show that CDH13 co-immunoprecipitates with ITGβ1 and ITGβ3, and that CDH13 has the ability to bind both ITGβ1 and ITGβ3 in the cell adhesion assay. Interestingly, while co-expression of GABAAα1/β3 did not affect the interaction between CDH13 and ITGβ1, co-expression of GABAAα1/β3 with ITGβ3 completely abolished the interaction between CDH13 and ITGβ3. Both, ITGβ1 and ITGβ3, are expressed by pyramidal neurons [47, 48], and we show that these are expressed postsynaptically together with GABAAα1. This points to the intriguing possibility that ITGβ1 and ITGβ3 could function as a molecular switch for synapse maintenance. A similar function for ITGβ1/ITGβ3 has already been described previously in spinal cord neurons, where these integrins have opposite functions in the regulation of synaptic dwell time of glycine receptors through stabilization (ITGβ1) and destabilization (ITGβ3) of the inhibitory synaptic scaffold protein gephyrin [46], and via this mechanism regulate the strength of glycinergic synapses. The differential function of ITGβ1/ITGβ3 would allow glutamatergic neurons to control the amount of inhibitory input they receive. Since both ITGβ1 and ITGβ3 are also expressed in glutamatergic synapses, ITGβ1/ITGβ3 might be in the ideal position to maintain the E/I balance by regulating simultaneously the E and I input, respectively, by stabilizing the excitatory and inhibitory postynaptic receptors [47, 48, 70]. It has recently been shown that cortical pyramidal neurons receive an amount of inhibitory synaptic input from GABAergic PV^+^ neurons that is corresponding relatively to the excitatory drive onto that pyramidal neuron, thereby maintaining their E/I balance [71]. Since individual PV^+^ GABAergic neurons can differentially regulate their inhibitory strength onto individual postsynaptic pyramidal neurons [71], it is likely that pyramidal neurons instruct the regulation of inhibitory synapses onto themselves. The complex of CDH13, ITGβ1, and ITGβ3 could play a role in this regulation. Loss of CDH13 would lead to the inability of the postsynaptic glutamatergic neuron to regulate inhibitory synapses formed onto itself via regulation of the ITGβ1/ITGβ3 ratio. Indeed, in *Cdh13*^*−/−*^ mice, we previously reported an increase in inhibitory synapses [9]. The importance of CDH13 in this mechanism is underlined by our finding that while Echistatin affected neuronal network activity of control networks, it has no effect in CDH13-deficient networks. ITGβ1 is known to interact with other Cadherin family members as well, such as Cdh5 in the mouse retinal vasculature [72]. Interestingly, a recent study used Proximity Labeling, Mass-Spectometry, and Atomic Force Microscopy to show that ITGβ1 binds specifically to the EC2 domain of CDH1 in a cell model [73]. CDH13 also contains an EC2 domain, which is used in an alternative non-strand swapping binding pattern when forming CDH13 homodimers [18]. Investigating whether the ITGβ1/ CDH13 interaction we showed here is realized via the same EC2 domain will be an interesting topic for future study.

## Methods

### Neuronal differentiation

HiPSCs from control #1, control #2, and control #6 were differentiated into Glutamatergic cortical layer 2/3 neurons by overexpressing mouse neuronal determinant Neurogenin 2 (*Ngn2*) upon doxycycline treatment [33] (referred to as iGLU#1-#3). GABAergic neurons were derived by overexpressing mouse neuronal determinant Achaete-scute homolog 1 (*Ascl1*, plasmid was custom designed and cloned by VectorBuilder and is available upon request) upon doxycycline treatment with supplementation of FSK (10 µM, Sigma). GABAergic neurons were generated from control hiPSC line #2-#6, referred to as iGABA#1-#5. From control #6, a CDH13 KO line was generated as described previously [43] and differentiated into GABAergic neurons (iGABA#5-KO). See Supplementary methods for further information on hiPSCs used in this study. Glutamatergic neurons were either cultured alone or in co-culture with iGABA_A-FSK_. When co-cultured, GABAergic neurons were plated at days in vitro (DIV) 0 and labeled with AAV2-hSyn-mCherry (UNC Vector Core) for visualization, with AAV2-hSyn-hChR2(H134R)-mCherry (UNC Vector Core) for optogenetic activation, or with lentivirus expressing GFP empty vector (control) or *CDH13*-shRNA (See Supplementary methods). After 5 h of incubation, cultures were washed twice with DMEM/F12 (Thermo Fisher Scientific) before iGLU_Ngn2_ were plated on top. When changing the E/I ratio from 95:5, 85:15, 75:25 to 65:35, the number of iGLU_Ngn2_ present in the culture was always kept at a similar density whereas the number of iGABA_A-FSK_ was increased to make sure baseline electrophysiological activity was kept constant. HiPSCs were plated in E8 flex supplemented with doxycycline (4 µg/ml), Revitacell (1:100, Thermo Fisher Scientific), and FSK. At DIV 1, cultures were switched to DMEM/F12 containing FSK (10 µM, Sigma), N2 (1:100, Thermo Fisher Scientific), non-essential amino acids (1:100, Sigma), primocin (0.1 µg/ml), NT3 (10 ng/ml), BDNF (10 ng/ml), and doxycycline (4 µg/ml). To support neuronal maturation, freshly prepared rat astrocytes [62] were added to the culture in a 1:1 ratio at DIV 2. At DIV 3, the medium was changed to Neurobasal medium (Thermo Fisher Scientific) supplemented with FSK (10 µM, Sigma), B-27 (Thermo Fisher Scientific), glutaMAX (Thermo Fisher Scientific), primocin (0.1 µg/ml), NT3 (10 ng/ml), BDNF (10 ng/ml), and doxycycline (4 µg/ml). Moreover, cytosine-b-D-arabinofuranoside (Ara-C; 2 µM; Sigma) was added once to remove any proliferating cell from the culture. From DIV 6 onwards, half of the medium was refreshed three times a week. The medium was additionally supplemented with 2.5% FBS (Sigma) to support astrocyte viability from DIV 10 onwards. After DIV 13, FSK and doxycycline were removed from the culture medium. Neuronal cultures were kept through the whole differentiation process at 37 °C/ 5% CO_2_. All experiments in Figs. 1–3 were performed using iGLU#1 + iGABA#1 or iGLU#1 + iGABA#2. We found no significant differences between the network activity on MEA, single-cell recordings nor immunohistochemistry (see Supplementary methods and Supplementary Table 10) analysis between these two E/I compositions, therefore all data were pooled in the respective analysis. All experiments including *CDH13*-shRNAs in Figs. 4 and 5 were performed using iGLU#1 + iGABA#1 or iGLU#2 + iGABA#1. Similarly, no significant differences were observed between both compositions before pooling the data. To validate the line-to-line variability amongst *Ascl1*-stable lines, we co-cultured all iGABA neurons (i.e., iGABA#1-#5) with iGLU#2 on MEA in Supplementary Fig. 1. E/I networks containing iGABA#5 and iGABA#5-KO in Figs. 4, 5 and Supplementary Fig. 5, we co-cultured with iGLU#3.

### Micro-electrode array recordings and data analysis

All recordings were performed using the 24-well MEA system (Multichannel Systems, MCS GmbH, Reutlingen, Germany) as described before [62, 67]. Spontaneous electrophysiological activity of E/I networks was recorded for 10 min at 37 °C and constant flow of humidified gas (5% CO_2_ and 95% O_2_). The raw signal was sampled at 10 kHz and filtered with a high-pass filter (i.e., 2nd-order Butterworth, 100-Hz cutoff frequency) and a low-pass filter (i.e., 4th-order Butterworth, 3500-Hz cutoff frequency). The threshold for detecting spikes was set at ±4.5 standard deviations. We performed off-line data analysis by using Multiwell Analyzer (i.e., software from the 24-well MEA system that allows the extraction of the spike trains) and in-house algorithms in MATLAB (The Mathworks, Natick, MA, USA) that allows the extraction of MEA parameters from multiwell analyzer, and parameters describing the burst shape. The parameters extracted using Multiwell analyzer in this paper include: the MFR (spikes/second in Hz. The MFR is averaged per well for all electrodes), the PRS (% spikes not included in the burst, nor network burst), the NBR (network burst/min), and duration (NBD; ms). We detected bursts per electrode based on the maximum inter-spike interval (ISI) of 30 ms to start or end a burst. If the ISI is shorter than 30 ms, spikes were included in the burst, if the ISI is larger than 30 ms the burst ends. All bursts that were <65 ms apart were merged. All bursts that have a duration of <50 ms or have <4 spikes were removed from the analysis. When a burst occurs simultaneously in more than 80% of the active channels, this is called a network burst. Discriminant functions are based on the following network activity parameters: firing rate, single channel burst rate, -duration, -firing rate in burst and -IBI, NBR, -duration and -IBI, PRS and was performed in SPSS (IBM Corporation, Armonk, NY, USA). Ellipses are centered on the group centroids.

In order to ensure, only mature and stable networks were included in analysis, we used the following exclusion criteria: not active wells (i.e., MFR > 0.1 Hz in at least three channels to be called active), we excluded controls wells only with a MFR < 1 Hz [67], wells in which <80% of the channels detected spikes, wells that showed no network bursts at DIV 28, wells where network bursts were detected in <80% of the channels, and wells where the firing rate decreased over development were rigorously discarded [67]. For further recommendations on optimal data analysis and experimental design of MEA experiments, see ref. [67].

### Single-cell electrophysiology

Coverslips were placed in the recording chamber of the electrophysiological setup, continuously perfused with oxygenated (95% O_2_/5% CO_2_) ACSF at 32 °C as described previously [62]. Patch pipettes with filament (6–8 MΩ) were pulled from borosilicate glass (Science Products GmbH, Hofheim, Germany) using a Narishige PC-10 micropipette puller (Narishige, London, UK). For all recordings of intrinsic properties and spontaneous activity, a potassium-based solution containing was used as described before [62]. vRMP was measured immediately after generation of a whole-cell configuration. Further analysis of active and passive membrane properties was conducted at a holding potential of −60 mV. Passive membrane properties were determined via voltage step of −10 mV. Active intrinsic properties were measured with a stepwise current injection protocol. Spontaneous activity was measured at either −60 mV (sPSCs, drug free or sIPSCs,100 µM CNQX, Tocris) or +10 mV (sIPSCs, 100 µM CNQX) at DIV 28, 35, and 49. Cells were visualized with an Olympus BX51WI upright microscope (Olympus Life Science, PA, USA), equipped with a DAGE-MTI IR−1000E (DAGE-MTI, IN, USA) camera) and a CoolLED PE-200 LED system (Scientifica, Sussex, UK) for fluorescent identification. A Digidata 1440-A digitizer and a Multiclamp 700B amplifier (Molecular Devices) were used for data acquisition. Sampling rate was set at 20 kHz and a low-pass 1-kHz filter was used during recording. Recordings were not corrected for liquid junction potential (±10 mV). Recordings were discarded if series resistance reached >25 MΩ or dropped below a 10:0 ratio of membrane resistance to series resistance. Intrinsic electrophysiological properties were analyzed using Clampfit 10.7 (molecular devices, CA, USA), and sPSCs were analyzed using MiniAnalysis 6.0.2 (Synaptosoft Inc, GA, USA) as previously described [62].

For the determination of decay times, GABAergic events were isolated in neurons at DIV 49 by bath application of CNQX. This decay time was then compared to the decay time of glutamatergic events recorded in the presence of PTX. We determined that a cutoff of 3.8 ms (Supplementary Fig. 2s) could to a high degree of confidence separate glutamatergic and GABAergic events in other data. This cutoff was then used to separate glutamatergic and GABAergic events during development.

### Cell adhesion assay

The cell aggregation assay was performed as described previously [50]. In brief, HEK293T cells were transfected with indicated constructs via calcium phosphate transfection when they reached a confluence of 50%. In case the transfection rate was above 75% after 26 h, aggregation assays were performed. Cells were trypsinized and collected by centrifugation for 5 min at 4 °C and 1000 rpm and washed once with serum-free medium, before being resuspended by pipetting in Hank´s Balanced Saline Solution (HBSS) (55 mM NaCl, 40 mM KCl, 15 mM MgSO_4_, 10 mM CaCl_2_, 20 mM glucose, 50 mM sucrose, 2 mg/ml bovine serum albumin, and 20 mM Tricine, pH 6.95). Cells were resuspended in HBSS to a final concentration of 1.2 × 10^6^ cells/ml for single line experiments, or 6 × 10^5^ when two different cell lines were incubated. One milliliter cell suspension was collected into 1.5 ml Eppendorf tubes and incubated at 4 °C under gentle agitation for 1 h. Aggregation was quantified by counting the cells with a hemocytometer and plotted as the ratio *T*0/*T*60 (*T0* = # of cellular particles before incubation, *T*60 = # cellular particles after 1-h incubation. Cellular aggregates count as single particles).

### Statistics

The statistical analysis for all experiments was performed using GraphPad Prism 8 (GraphPad Software, Inc., CA, USA). We ensured normal distribution using a Kolmogorov–Smirnov normality test. To determine statistical significance for the different experimental conditions, *p* values < 0.05 were considered to be significant. Statistical analysis was performed with one-way ANOVA and post hoc Tukey (normal distribution; Fig. 1), or Kruskal–Wallis ANOVA with post hoc Dunn’s correction for multiple testing (not normally distributed data; Fig. 3d and Supplementary Figs. 1e–h, 2a–p, t, u, 4c–e, 5c, e, i). Statistical analysis over development (Fig. 2) was performed with Two-ways ANOVA and Post hoc Bonferroni (normal distribution) or a Mixed effect analysis and post hoc Dunn’s (not normally distributed) correction for multiple testing (depending on normal distribution). When comparing means of two variables at one individual time-point, we used a paired *T*-test (paired data; Figs. 3g–k, 5r and Supplementary Figs. 1i, j, 4b, f–j, 5m) or Mann–Whitney *U*-test (unpaired data; Figs. 4b–d, f, j, 5i–p and Supplementary Figs. 2q, s, 3c, 5d, j), and if applicable, corrected for multiple testing using Bonferroni. Nested One-Way ANOVA with post hoc Sidak correction was performed on normalized NKCC1 and KCC2 data in Supplementary Fig. 3f. Statistics on histograms were performed using Multiple *t*-test on bins using the Holm–Sidak method (Figs. 3c, h–k, 4g, k and Supplementary Fig. 5f). Statistics on cumulative distribution were performed with a Kolmogorov–Smirnov test (Fig. 4d and Supplementary Fig. 5i, j). Data are presented as mean ± standard error of the mean and reported in Supplementary Tables 2–8 and 10–12.

## Supplementary information

supplementary material

Supplementary table 1

## Data Availability

MEA data were analyzed using Multiwell Analyzer software (Multichannel Systems) and a custom-made in-house code developed in MATLAB (The Mathworks, Natick, MA, USA, 2018) for the extraction of parameters describing spontaneous network activity (available upon request). The generation of average burst shapes was performed using previously published scripts and functions implemented in MATLAB [74].

## References

[1] He HY, Shen W, Zheng L, Guo X, Cline HT (2018). Excitatory synaptic dysfunction cell-autonomously decreases inhibitory inputs and disrupts structural and functional plasticity. Nat Commun.

[2] Sohal VS, Rubenstein JLR (2019). Excitation-inhibition balance as a framework for investigating mechanisms in neuropsychiatric disorders. Mol Psychiatry.

[3] Zoghbi HY, Bear MF (2012). Synaptic dysfunction in neurodevelopmental disorders associated with autism and intellectual disabilities. Cold Spring Harb Perspect Biol.

[4] Selten M, van Bokhoven H, Nadif, Kasri N (2018). Inhibitory control of the excitatory/inhibitory balance in psychiatric disorders. F1000Res.

[5] Dani VS, Chang Q, Maffei A, Turrigiano GG, Jaenisch R, Nelson SB (2005). Reduced cortical activity due to a shift in the balance between excitation and inhibition in a mouse model of Rett Syndrome. Proc Natl Acad Sci USA.

[6] Calfa G, Li W, Rutherford JM, Pozzo-Miller L (2015). Excitation/inhibition imbalance and impaired synaptic inhibition in hippocampal area CA3 of Mecp2 knockout mice. Hippocampus.

[7] Bateup HS, Johnson CA, Denefrio CL, Saulnier JL, Kornacker K, Sabatini BL (2013). Excitatory/inhibitory synaptic imbalance leads to hippocampal hyperexcitability in mouse models of tuberous sclerosis. Neuron.

[8] Gao R, Penzes P (2015). Common mechanisms of excitatory and inhibitory imbalance in schizophrenia and autism spectrum disorders. Curr Mol Med.

[9] Rivero O, Selten MM, Sich S, Popp S, Bacmeister L, Amendola E (2015). Cadherin-13, a risk gene for ADHD and comorbid disorders, impacts GABAergic function in hippocampus and cognition. Transl Psychiatry.

[10] Sanders SJ, He X, Willsey AJ, Ercan-Sencicek AG, Samocha KE, Cicek AE (2015). Insights into autism spectrum disorder genomic architecture and biology from 71 risk loci. Neuron.

[11] Asherson P, Zhou K, Anney RJ, Franke B, Buitelaar J, Ebstein R (2008). A high-density SNP linkage scan with 142 combined subtype ADHD sib pairs identifies linkage regions on chromosomes 9 and 16. Mol Psychiatry.

[12] Neale BM, Lasky-Su J, Anney R, Franke B, Zhou K, Maller JB (2008). Genome-wide association scan of attention deficit hyperactivity disorder. Am J Med Genet B Neuropsychiatr Genet.

[13] Neale BM, Medland SE, Ripke S, Asherson P, Franke B, Lesch KP (2010). Meta-analysis of genome-wide association studies of attention-deficit/hyperactivity disorder. J Am Acad Child Adolesc Psychiatry.

[14] Mavroconstanti T, Johansson S, Winge I, Knappskog PM, Haavik J (2013). Functional properties of rare missense variants of human CDH13 found in adult attention deficit/hyperactivity disorder (ADHD) patients. PLoS ONE.

[15] Howard DM, Adams MJ, Clarke T-K, Hafferty JD, Gibson J, Shirali M (2019). Genome-wide meta-analysis of depression identifies 102 independent variants and highlights the importance of the prefrontal brain regions. Nat Neurosci.

[16] Johnson C, Drgon T, Liu QR, Walther D, Edenberg H, Rice J (2006). Pooled association genome scanning for alcohol dependence using 104,268 SNPs: validation and use to identify alcoholism vulnerability loci in unrelated individuals from the collaborative study on the genetics of alcoholism. Am J Med Genet B Neuropsychiatr Genet.

[17] Treutlein J, Cichon S, Ridinger M, Wodarz N, Soyka M, Zill P (2009). Genome-wide association study of alcohol dependence. Arch Gen Psychiatry.

[18] Ciatto C, Bahna F, Zampieri N, VanSteenhouse HC, Katsamba PS, Ahlsen G (2010). T-cadherin structures reveal a novel adhesive binding mechanism. Nat Struct Mol Biol.

[19] Ranscht B, Dours-Zimmermann MT (1991). T-cadherin, a novel cadherin cell adhesion molecule in the nervous system lacks the conserved cytoplasmic region. Neuron.

[20] Joshi MB, Ivanov D, Philippova M, Erne P, Resink TJ (2007). Integrin-linked kinase is an essential mediator for T-cadherin-dependent signaling via Akt and GSK3beta in endothelial cells. FASEB J.

[21] Forero A, Ku HP, Malpartida AB, Waldchen S, Alhama-Riba J, Kulka C (2020). Serotonin (5-HT) neuron-specific inactivation of Cadherin-13 impacts 5-HT system formation and cognitive function. Neuropharmacology.

[22] Fredette BJ, Miller J, Ranscht B (1996). Inhibition of motor axon growth by T-cadherin substrata. Development.

[23] Killen AC, Barber M, Paulin JJW, Ranscht B, Parnavelas JG, Andrews WD (2017). Protective role of Cadherin 13 in interneuron development. Brain Struct Funct.

[24] Tantra M, Guo L, Kim J, Zainolabidin N, Eulenburg V, Augustine GJ (2018). Conditional deletion of Cadherin 13 perturbs Golgi cells and disrupts social and cognitive behaviors. Genes Brain Behav.

[25] Rivero O, Sich S, Popp S, Schmitt A, Franke B, Lesch KP (2013). Impact of the ADHD-susceptibility gene CDH13 on development and function of brain networks. Eur Neuropsychopharmacol.

[26] Paradis S, Harrar DB, Lin Y, Koon AC, Hauser JL, Griffith EC (2007). An RNAi-based approach identifies molecules required for glutamatergic and GABAergic synapse development. Neuron.

[27] Ferguson BR, Gao WJ (2018). PV interneurons: critical regulators of E/I balance for prefrontal cortex-dependent behavior and psychiatric disorders. Front Neural Circuits.

[28] Scheyltjens I, Arckens L (2016). The current status of somatostatin-interneurons in inhibitory control of brain function and plasticity. Neural Plast.

[29] Sun AX, Yuan Q, Tan S, Xiao Y, Wang D, Khoo AT (2016). Direct induction and functional maturation of forebrain GABAergic neurons from human pluripotent stem cells. Cell Rep.

[30] Yang N, Chanda S, Marro S, Ng YH, Janas JA, Haag D (2017). Generation of pure GABAergic neurons by transcription factor programming. Nat Methods.

[31] Yuan F, Chen X, Fang KH, Wang Y, Lin M, Xu SB (2018). Induction of human somatostatin and parvalbumin neurons by expressing a single transcription factor LIM homeobox 6. Elife.

[32] Filice F, Schwaller B, Michel TM, Grünblatt E (2020). Profiling parvalbumin interneurons using iPSC: challenges and perspectives for Autism Spectrum Disorder (ASD). Mol Autism.

[33] Zhang Y, Pak C, Han Y, Ahlenius H, Zhang Z, Chanda S (2013). Rapid single-step induction of functional neurons from human pluripotent stem cells. Neuron.

[34] Shi Z, Zhang J, Chen S, Li Y, Lei X, Qiao H (2016). Conversion of fibroblasts to parvalbumin neurons by one transcription factor, Ascl1, and the chemical compound forskolin. J Biol Chem.

[35] Ito-Ishida A, Ure K, Chen H, Swann JW, Zoghbi HY (2015). Loss of MeCP2 in parvalbumin-and somatostatin-expressing neurons in mice leads to distinct Rett syndrome-like phenotypes. Neuron.

[36] Medrano-Fernandez A, Delgado-Garcia JM, Del Blanco B, Llinares M, Sanchez-Campusano R, Olivares R (2019). The epigenetic factor CBP is required for the differentiation and function of medial ganglionic eminence-derived interneurons. Mol Neurobiol.

[37] Mayer C, Hafemeister C, Bandler RC, Machold R, Batista Brito R, Jaglin X (2018). Developmental diversification of cortical inhibitory interneurons. Nature.

[38] Sommeijer JP, Levelt CN (2012). Synaptotagmin-2 is a reliable marker for parvalbumin positive inhibitory boutons in the mouse visual cortex. PLoS ONE.

[39] Favuzzi E, Deogracias R, Marques-Smith A, Maeso P, Jezequel J, Exposito-Alonso D (2019). Distinct molecular programs regulate synapse specificity in cortical inhibitory circuits. Science.

[40] Vardi R, Goldental A, Sardi S, Sheinin A, Kanter I (2016). Simultaneous multi-patch-clamp and extracellular-array recordings: single neuron reflects network activity. Sci Rep.

[41] Ben-Ari Y (2002). Excitatory actions of gaba during development: the nature of the nurture. Nat Rev Neurosci.

[42] Chubykin AA, Atasoy D, Etherton MR, Brose N, Kavalali ET, Gibson JR (2007). Activity-dependent validation of excitatory versus inhibitory synapses by neuroligin-1 versus neuroligin-2. Neuron.

[43] Vitale MR, Zöller JEM, Jansch C, Janz A, Edenhofer F, Klopocki E (2021). Generation of induced pluripotent stem cell lines deficient for Cadherin 13 (UKWMPi002-A-1/B/C) Bassociated with neurodevelopmental disorders using CRISPR/Cas9. Stem Cell Res.

[44] Philippova M, Ivanov D, Joshi MB, Kyriakakis E, Rupp K, Afonyushkin T (2008). Identification of proteins associating with glycosylphosphatidylinositol-anchored T-cadherin on the surface of vascular endothelial cells: role for Grp78/BiP in T-cadherin-dependent cell survival. Mol Cell Biol.

[45] Mukoyama Y, Utani A, Matsui S, Zhou S, Miyachi Y, Matsuyoshi N (2007). T-cadherin enhances cell-matrix adhesiveness by regulating beta1 integrin trafficking in cutaneous squamous carcinoma cells. Genes Cells.

[46] Charrier C, Machado P, Tweedie-Cullen RY, Rutishauser D, Mansuy IM, Triller A (2010). A crosstalk between beta1 and beta3 integrins controls glycine receptor and gephyrin trafficking at synapses. Nat Neurosci.

[47] Chan C-S, Weeber EJ, Zong L, Fuchs E, Sweatt JD, Davis RL (2006). β1-integrins are required for hippocampal AMPA receptor-dependent synaptic transmission, synaptic plasticity, and working memory. J Neurosci.

[48] Pozo K, Cingolani LA, Bassani S, Laurent F, Passafaro M, Goda Y (2012). beta3 integrin interacts directly with GluA2 AMPA receptor subunit and regulates AMPA receptor expression in hippocampal neurons. Proc Natl Acad Sci U S A.

[49] Klausberger T, Roberts JD, Somogyi P (2002). Cell type- and input-specific differences in the number and subtypes of synaptic GABA(A) receptors in the hippocampus. J Neurosci.

[50] Nguyen T, Sudhof TC (1997). Binding properties of neuroligin 1 and neurexin 1beta reveal function as heterophilic cell adhesion molecules. J Biol Chem.

[51] Katsamba P, Carroll K, Ahlsen G, Bahna F, Vendome J, Posy S (2009). Linking molecular affinity and cellular specificity in cadherin-mediated adhesion. Proc Natl Acad Sci U S A.

[52] Shapiro L, Fannon AM, Kwong PD, Thompson A, Lehmann MS, Grubel G (1995). Structural basis of cell–cell adhesion by cadherins. Nature.

[53] Tretter V, Ehya N, Fuchs K, Sieghart W (1997). Stoichiometry and assembly of a recombinant GABAA receptor subtype. J Neurosci.

[54] Weitzman JB, Chen A, Hemler ME (1995). Investigation of the role of beta 1 integrins in cell-cell adhesion. J Cell Sci.

[55] Pfaff M, McLane M, Beviglia L, Niewiarowski S, Timpl R (2009). Comparison of disintegrins with limited variation in the RGD loop in their binding to purified integrins αIIbβ3, αVβ3 and α5β1 and in cell adhesion inhibition. Cell Adhes Commun.

[56] Geissler J, Lesch KP (2011). A lifetime of attention-deficit/hyperactivity disorder: diagnostic challenges, treatment and neurobiological mechanisms. Expert Rev Neurother.

[57] Rubenstein JL, Merzenich MM (2003). Model of autism: increased ratio of excitation/inhibition in key neural systems. Genes Brain Behav.

[58] Nicholas CR, Chen J, Tang Y, Southwell DG, Chalmers N, Vogt D (2013). Functional maturation of hPSC-derived forebrain interneurons requires an extended timeline and mimics human neural development. Cell Stem Cell.

[59] Ben-Ari Y, Gaiarsa JL, Tyzio R, Khazipov R (2007). GABA: a pioneer transmitter that excites immature neurons and generates primitive oscillations. Physiol Rev.

[60] Sahasranamam A, Vlachos I, Aertsen A, Kumar A (2016). Dynamical state of the network determines the efficacy of single neuron properties in shaping the network activity. Sci Rep.

[61] Baltz T, de Lima AD, Voigt T (2010). Contribution of GABAergic interneurons to the development of spontaneous activity patterns in cultured neocortical networks. Front Cell Neurosci.

[62] Frega M, Linda K, Keller JM, Gümüş-Akay G, Mossink B, van Rhijn J-R (2019). Neuronal network dysfunction in a model for Kleefstra syndrome mediated by enhanced NMDAR signaling. Nat Commun.

[63] Odawara A, Katoh H, Matsuda N, Suzuki I (2016). Physiological maturation and drug responses of human induced pluripotent stem cell-derived cortical neuronal networks in long-term culture. Sci Rep.

[64] Teppola H, Acimovic J, Linne ML (2019). Unique features of network bursts emerge from the complex interplay of excitatory and inhibitory receptors in rat neocortical networks. Front Cell Neurosci.

[65] Jimbo Y, Kawana A, Parodi P, Torre V (2000). The dynamics of a neuronal culture of dissociated cortical neurons of neonatal rats. Biol Cybern.

[66] Suresh J, Radojicic M, Pesce LL, Bhansali A, Wang J, Tryba AK (2016). Network burst activity in hippocampal neuronal cultures: the role of synaptic and intrinsic currents. J Neurophysiol.

[67] Mossink B, Verboven AHA, van Hugte EJH, Gunnewiek TMK, Parodi G, Linda K, et al. Human neuronal networks on micro-electrode arrays are a highly robust tool to study disease-specific genotype-phenotype correlations in vitro. bioRxiv. 2021. Preprint at 10.1101/2021.1101.1120.427439. 10.1016/j.stemcr.2021.07.001 PMC8452490 34329594

[68] Bradley JA, Luithardt HH, Metea MR, Strock CJ (2018). In vitro screening for seizure liability using microelectrode Array technology. Toxicol Sci.

[69] Tukker AM, Wijnolts FMJ, de Groot A, Westerink RHS (2020). Applicability of hiPSC-derived neuronal cocultures and rodent primary cortical cultures for in vitro seizure liability assessment. Toxicol Sci.

[70] Park YK, Goda Y (2016). Integrins in synapse regulation. Nat Rev Neurosci.

[71] Xue M, Atallah BV, Scanziani M (2014). Equalizing excitation-inhibition ratios across visual cortical neurons. Nature.

[72] Yamamoto H, Ehling M, Kato K, Kanai K, van Lessen M, Frye M (2015). Integrin β1 controls VE-cadherin localization and blood vessel stability. Nat Commun.

[73] Shafraz O, Xie B, Yamada S, Sivasankar S (2020). Mapping transmembrane binding partners for E-cadherin ectodomains. Proc Natl Acad Sci.

[74] Van De Vijver S, Missault S, Van Soom J, Van Der Veken P, Augustyns K, Joossens J (2019). The effect of pharmacological inhibition of Serine Proteases on neuronal networks in vitro. PeerJ.

