# Supplementary material for: Cadherin-13 is a critical regulator of GABAergic modulation in human stem-cell-derived neuronal networks
Source: Mol Psychiatry. 2021 May 10;27(1):1–18. doi: 10.1038/s41380-021-01117-x (PMC8960401; doi:10.1038/s41380-021-01117-x)
Supplement: Supplementary file 1 — supplementary material [file 41380_2021_1117_MOESM1_ESM.pdf]

Cadherin-13 is a critical regulator of GABAergic modulation in human stem cell derived neuronal networks

Supplementary Methods

- Cell line information and hiPSC generation
- RNA interference
- Optogenetics
- Animals
- Compound application
- Immunocytochemistry
- Quantification of mRNA by RT-qPCR
- RNA-sequencing
- Pre-processing of RNA-seq data
- IP and Western blot

Supplementary Figures

- **Supplementary figure 1**: *Ascl1* overexpression robustly generates GABAergic neurons from several hiPSC lines that shape neuronal network activity
- **Supplementary figure 2**: $iGLU_{Ngn2}$ and $iGABA_{A\text{-}FSK}$ mature over development
- **Supplementary figure 3**: $iGABA_{A\text{-}FSK}$ become functionally inhibitory at DIV 49
- **Supplementary figure 4**: Functional GABAergic modulation in E/I networks is depending on the hyperpolarizing GABA shift and scalable to the percentage $iGABA_{A\text{-}FSK}$ present in the network.
- **Supplementary figure 5**: E/I cultures pose a valid model to study cell-type specific interactions of NDD genes to network dysfunction; an example for CDH13-deficiency

Supplementary Tables

- **Supplementary table 1**: Count table with raw counts/(log)cpm values displayed in **Figure 1f** and **Supplementary figure 5a**.
- **Supplementary table 2**: Statistics of intrinsic properties from E/I networks in **Figure 1** and **Supplementary figure 2**.
- **Supplementary table 3**: Statistics of **Supplementary figure 3c, f**
- **Supplementary table 4**: Statistics of **Figure 2g-j**
- **Supplementary table 5**: Statistics of **Supplementary figure 1e, h-j**
- **Supplementary table 6**: Statistics of **Figure 3d** and **Supplementary figure 4c-e**
- **Supplementary table 7**: Statistics of **Figure 3g, j and k and Supplementary figure 4b, f-j**

- **Supplementary table 8**: Statistics of **Supplementary figure 5c**
- **Supplementary table 9:** Forward and reverse primer sequences, *CDH13* targeting short hairpin RNA sequences.
- **Supplementary table 10**: Statistics of **Figure 4d, f, j and Supplementary figure 5e, i, j**
- **Supplementary table 11**: Statistics of **Figure 5i-p**
- **Supplementary table 12**: Statistics of **Figure 5r and Supplementary figure 5m**
- **Supplementary table 13**: Used primary and secondary antibodies

Supplementary Methods

Cell line information and hiPSC generation

In this study we used in total 6 control hiPSC lines, Control #1-6. All hiPSCs used in this study were obtained from reprogrammed fibroblasts. Control line 1 was obtained from a healthy 30-year-old female, and reprogrammed via episomal reprogramming[1]. Control line 2 was derived from a healthy 51-year-old male and reprogrammed via a non-integrating Sendai virus by KULSTEM (Leuven, Belgium). Control line 3 was obtained from a healthy male, and reprogrammed via retroviral vectors expressing four transcription factors: Oct4, Sox2, Klf4, and cMyc. Generated clones (at least two per patient line) were selected and tested for pluripotency and genomic integrity based on single nucleotide polymorphism (SNP) arrays[2]. Control line 4 was derived from a curated control cell line[3, 4]. This control line originated from a 36-year old female, reprogrammed using episomal vector-based reprogramming of the Yamanaka transcription factors *Oct4*, *c-Myc*, *Sox2* and *Klf4*[5], showing no karyotypical malformations. Control line 5 was derived from a 30-year old male[6, 7] and reprogrammed using episomal vector-based reprogramming of the Yamanaka factors and was tested for genetic integrity using SNP assay[8]. Control line 6 was derived from a 46-year old female and reprogrammed via non-integrative Sendai virus (CytoTune-iPS Reprogramming Kit 2.0 (Thermo Fisher))[9]. From control line 6 a CDH13 knockout (KO) line was generated as described previously[9]. PCR Cloning (NEB) and subsequent DNA sequencing (LGC genomics) revealed that the *CDH13*$^{-/-}$ hiPSC line carries one allele with a deletion of four nucleotides and the second allele with an insertion of one nucleotide. Control line 6 and *CDH13*$^{-/-}$ hiPSC were investigated for the expression of germ layer markers (α-SMA, AFP and β-Tubulin) and pluripotency markers (OCT3/4, SSEA-4, TRA-1-60) via immunofluorescence and qRT-PCR. Standard G-banding revealed no numerical or structural chromosome abnormalities. The absence of Sendai virus specific transcripts was confirmed by RT-PCR. Short tandem repeat (STR) analysis showed that parental fibroblasts and newly created hiPSCs shared alleles with a 100% match[9]. All hiPSCs were regularly tested for mycoplasma contamination. HiPSCs were cultured on Matrigel (Corning, #356237) in E8 flex (Thermo Fisher Scientific) supplemented with primocin (0.1 µg/ml, Invivogen) and low puromycin (0.5 µg/ml, to select for rtTA positive cells) and G418 concentrations (50 µg/ml, to select for *Ngn2* or *Ascl1*

positive cells) at 37˚C/5% CO_2. Medium was refreshed every 2-3 days and cells were passaged twice a week using an enzyme-free dissociation reagent (ReLeSR, Stem Cell Technologies).

RNA interference

For RNAi knockdown experiments, DNA fragments encoding shRNAs directed against human *CDH13* mRNA (Sigma, Supplementary table 8 and 9) were cloned into the pTRIPΔU3-EF1α-EGFP lentiviral vector. Empty vector expressing GFP only was used as control vector. Lentiviral particles were prepared from both shRNA expressing vectors and empty vector, and tittered as described previously[10].

Optogenetics

Optogenetic activation of E/I networks were performed at DIV 49 using the MW24-opto-stim LED cap for the Multiwell-MEA system (Multichannel Systems). Stimulation of cultures was conducted as follows: 200ms, 30.00mA, 470nm LED light pulses were delivered to each well. Inter stimulus interval was set at 5 seconds (onset to onset), and the stimulation protocol was repeated 24 times for a total duration of 2 minutes. Pre stimulation condition in Supplementary figure 4i and j represents the MFR normalized to 50 ms pre-stimulation baseline activity. Post stimulation condition represents the activity in a window between 10-30 ms after stimulus onset. Both pre and post stimulation responses on the level of the MFR were normalized to pre-stimulation condition.

Animals

The rodent astrocytes presented in this study were harvested embryonic (E18) rat brains (Wistar Wu) as previously described[2, 11, 12]. All experiments on animals were carried out in accordance with the approved animal care and use guidelines of the Animal Care Committee, Radboud University Medical Centre, the Netherlands, (RU-DEC-2011-021, protocol number: 77073).

Compound application

Picrotoxin (PTX, Tocris Cat No 1128) and Bicuculline (BIC, Sigma Cat No B6889) were prepared fresh into concentrated stocks and stored frozen at −20°C (PTX 50 mM in ETOH (MEA) or 100 mM in

DMSO (single-cell recordings); BIC 20 mM in DMSO). For all experiments on MEAs an aliquot of the concentrated stock PTX or BIC was first diluted 1:2 in room temperature DPBS and vortexed briefly. Then, 2.5 µl working dilution was added directly to the cell culture medium (500 µl) to reach a 100 µM concentration for PTX, and 40 µM concentration for BIC. ETOH or pre-diluted DMSO were used as vehicle. For all single cell experiments, PTX was directly diluted 1000 x in artificial cerebrospinal fluid (ACSF). The DMSO concentration in the ACSF was always ≤0.05% v/v. All experiments were performed at 37°C.

The GABA reversal was measured using a cesium-based intracellular solution containing (in mM) 115 $CsMeSO_3$, 20 CsCl, 10 HEPES, 2.5 $MgCl_2$, 4 Na_2ATP, 0.4 Na_3GTP, 10 sodium phosphocreatine, 0.6 EGTA (pH 7.2, mOsmol 290). For sucrose application, cells were recorded with the KCl based solution described before. GABA (10 mM dissolved in ACSF) was applied locally at a distance of 10-20 µm from the soma of the patched excitatory neuron using a PDES-2DX pressure ejection system (NPI, Tamm, Germany). Micropipettes used for compound application had a resistance of 3-5 MΩ. Injection pressure was set at 7psi/0.5 bar and injection duration was set to 100 ms. Analysis of peak response and reversal potential was conducted using Clampfit 10.7.

Immunocytochemistry

Cells were fixed and stained as described before[2]. All antibodies are listed in Supplementary table 13. Neurons were generally fixated at DIV 49, and at DIV 35 and DIV 49 for membrane expression of NKCC1 and KCC2. When membrane expression of NKCC1 and KCC2 was examined, coverslips were not permeabilized. We imaged at a 20x magnification to count the number of GABAergic subtypes and at a 63x magnification for all other measures using the Zeiss Axio Imager Z1 equipped with apotome. Images in figure 5 c and e were taken with the Zeiss AxioObserver Z1 with AryScan. All conditions within a batch were acquired with the same settings in order to compare signal intensities between different experimental conditions. Fluorescent signals were quantified using FIJI software. The intensity of NKCC1 or KCC2 expression on the cell membrane was calculated by: integrated density – (Area of selected cell X Mean fluorescence of background readings). The number of synaptic puncta was

determined per individual cell via manual counting and divided by the dendritic length of the dendrite. VGAT puncta intensity was determined using particle analysis in the FIJI software. All analysis was performed blinded for genotype using an open source random names application.

Quantification of mRNA by RT-qPCR

RNA samples were isolated using Nucleospin RNA isolation kit (Macherey-Nagel, 740955.250) according to the manufacturer's instructions. RNA samples were converted into cDNA by iScript cDNA synthesis kit (BIO-RAD, 1708891). CDNA products were cleaned up using the Nucleospin Gel and PCR clean-up kit (Macherey-Nagel, 740609.250). Human-specific primers were designed with Primer3plus (http://www.bioinformatics.nl/cgi-bin/primer3plus/primer3plus.cgi) and IDT PrimerQuest (https://eu.idtdna.com) tools, respectively. Primer sequences are given in supplementary table 9. QPCRs were performed in the Quantstudio 3 apparatus (Thermo Fisher Scientific) with GoTaq qPCR master mix 2X with SYBR Green (Promega, A600A) according to the manufacturer's protocol. The qPCR program was designed as following: After an initial denaturation step at 95°C for 10 min, PCR amplifications proceeded for 40 cycles of 95°C for 15 s and 60°C for 30 s and followed by a melting curve. All samples were analyzed in duplicate in the same run, placed in adjacent wells. The arithmetic mean of the Ct values of the technical replicas was used for calculations. Relative mRNA expression levels were calculated using the 2^-ΔΔCt method with standardization to housekeeping genes[13].

RNA-sequencing

RNA was isolated from three biological replicates of E/I networks composed of iGLU#2 and iGABA#1 (DIV 49) with the *Quick*-RNA Microprep kit (Zymo Research, R1051) according to manufacturer's instructions. RNA quality was checked using Agilent's Tapestation system (RNA High Sensitivity ScreenTape and Reagents, 5067-5579/80). RIN values ranged between 7.5 – 8.3. RNA-sequencing (RNA-seq) library preparation was performed using a published single-cell RNA-seq protocol from Cao et al. 2017[14] which was adapted for bulk RNA-seq experiments. For each sample, 10 ng total RNA (in 0.65 μL) was mixed with 0.1 μL dNTP mix (10 mM each) (Invitrogen, 10297018), 0.15 μL ERCC RNA Spike-In Mix (100.000x diluted) (Thermo Scientific, 4456740), 0.15 μL nuclease-free water (NF H2O)

and 0.4 μL anchored oligo-dT (2.5 μM) primer(5′-ACGACGCTCTTCCGATCTNNNNNNNN[10bp index]TTTTTTTTTTTTTTTTTTTTTTTTTTTTVN-3′, where "N" is any base and "V" is either "A", "C" or "G"; IDT) in a tube containing 7 μL Vapor-Lock (Qiagen, 981611) to prevent evaporation. Each sample was incubated for 5 min at 65°C and directly placed on ice. First strand reaction mix was added, consisting of 0.4 μL Maxima RT buffer (5X) (Thermo Scientific, EP0751), 0.05 μL RNasin Plus (Promega, N2611) and 0.1 μL Maxima H Minus Reverse Transcriptase (Thermo Scientific, EP0751). Reverse transcription was performed by incubating the samples at 50°C for 30 min and terminated by heating at 85°C for 5 min. For second strand synthesis, 2 μL RT product was mixed with 7.7 μL NF H2O, 2.5 μL Second Strand Buffer (Invitrogen, 10812014), 0.25 μL dNTP mix (10 mM each), 0.35 μL DNA polymerase I (*E. coli*) (NEB, M0209), 0.09 μL DNA ligase (*E. coli*) (NEB, M0205) and 0.09 μL Ambion RNase H (*E. coli*) (Invitrogen, AM2293). Second strand synthesis was performed by incubating samples at 16°C for 150 min, followed by 75°C for 20 min. Next, 0.5 μL Exonuclease I (NEB, M0293) was added per sample and incubated at 37°C for 60 min. cDNA samples were pooled per sets of 6-8 samples, Vapor-Lock was removed and samples were added up with NF H2O to a total volume of 107.6 μL. Each pool of samples was then purified using 79 μL beads buffer (20% PEG-8000 in 2.5 M NaCl, final concentrations) and 50 μL Ampure XP Beads (Beckman Coulter, A63881), and eluted in 7 μL NF H2O.

Tagmentation was performed per pool by adding 3 μL double-stranded cDNA sample to 5.5 μL Nextera TD buffer (Illumina, 15027866), 2.5 μL NF H2O and 1.0 μL TDE1 Enzyme (Illumina, 15027865), which was incubated at 55°C for 5 min. Samples were directly placed on ice for at least 3 min. The reaction was terminated by adding 12 μL Buffer PB (QiaQuick, 19066) and incubating for 5 min at room temperature. Samples were purified using 48 μL Ampure XP beads (Beckman Coulter, A63881) and eluted in 10 μL NF H2O. Next, each sample was mixed with 2 μL P5 primer (10 μM), (5′-AATGATACGGCGACCACCGAGATCTACAC[i5]ACACTCTTTCCCTACACGACGCTCTTC CGATCT-3′;IDT), 2 μL P7 primer (10 μM) (5′-CAAGCAGAAGACGGCATACGAGAT[i7] GTCTCGTGGGCTCGG-3′; IDT) and 20 μL NEBNext High-Fidelity 2X PCR Master Mix (NEB,

M0541). Amplification was performed using the following program: 72°C for 5 min, 98°C for 30 sec, 15 cycles of (98°C for 10 sec, 66°C for 30 sec, 72°C for 1 min) and a final step at 72°C for 5 min. Samples were purified using 32 µL Ampure XP beads (Beckman Coulter, A63881) and eluted in 12 µL NF H2O. Libraries were visualized by electrophoresis on a 1% agarose and 1X TAE gel containing 0.3 µg/mL ethidium bromide (Invitrogen, 15585011). Gel extraction was performed to select for products between 200 – 1000 bp using the Nucleospin Gel and PCR Clean-up kit (Macherey-Nagel, 740609). Samples were eluted in 11 µL NF H2O. cDNA concentrations were measured by Qubit dsDNA HS Assay kit (Invitrogen, Q32854). Product size distributions were visualized using Agilent's Tapestation system (D5000 ScreenTape and Reagents, 5067-5588/9). Libraries were sequenced on the NextSeq 500 platform (Illumina) using a V2 75 cycle kit (Read 1: 18 cycles, Read 2: 52 cycles, Index 1: 10 cycles).

Pre-processing of RNA-seq data

Base calls were converted to fastq format and demultiplexed using Illumina's bcl2fastq conversion software (v2.16.0.10) tolerating one mismatch per library barcode. Reads were filtered for valid unique molecular identifier (UMI) and sample barcode, tolerating one mismatch per barcode. Trimming of adapter sequences and over-represented sequences was performed using Trimmomatic (version 0.33)[15]. Trimmed reads were mapped to a combined human (GRCh38.p12) and rat (Rnor_6.0) reference genome to separate reads belonging to the human iNeurons from reads originating from the rat astrocytes. Mapping was performed using STAR[16] (version 2.5.1b) with default settings (--runThreadN 1, --outReadsUnmapped None, --outFilterType Normal, --outFilterScoreMin 0, --outFilterMultimapNmax 10, --outFilterMismatchNmax 10, --alignIntronMin 21, --alignIntronMax 0, --alignMatesGapMax 0, --alignSJoverhangMin 5, --alignSJDBoverhangMin 3, --sjdbOverhang 100). Uniquely mapped reads (mapping quality of 255) were extracted and read duplicates were removed using the UMI-tools software package[17]. Raw reads from BAM files were further processed to generate count matrices with HTSeq[18] (version 0.9.1) using reference transcriptome Gencode GRCh38.p12 (release 29, Ensembl 94). Raw counts were transformed to log-transformed counts per million (logCPM) using edgeR (R package). A

count table with raw counts/(log)cpm values can be found in supplementary table 1, and are deposited in GEO with the accession code GSE144197.

IP and Western blot

Protein extracts from iNeurons (lysis buffer 50 mM Tris-HCl pH 7.5, 1 mM EDTA pH 8.0, 150 mM NaCl, 1% Triton X-100, 2 mM CaCl2 and EDTA-free Protease Inhibitor Cocktail (Roche, 11836170001)) were incubated with 10 ug Anti-T-cadherin (CDH13) (Sigma-Aldrich, ABT121) or IgG and 1.5 mg Dynabeads™ Protein G (Invitrogen, 10007D) using the indirect technique. Input and IP samples were separated by SDS-PAGE, transferred on nitrocellulose membranes (BioRad 170-4158) and probed with antibodies against Integrin β1 (1:5000 Abcam, ab183666) and CDH13 (1:1000 Sigma-Aldrich, ABT121). Proteins were then detected with Horseradish Peroxidase conjugated Goat anti-Mouse (1:50.000 Jackson ImmunoResearch Laboratories, 115-035-062) and Goat anti-Rabbit (1:50.000 Invitrogen, G21234). Proteins were revealed with Super Signal West Femto ECL (Thermo Scientific, 34095) and visualized with ChemiDoc Touch Imaging system (BioRad).

Protein extracts from E/I networks (lysis buffer 50 mM Tris-HCl pH 7.5, 1 mM EDTA pH 8.0, 150 mM NaCl, 1% Triton X-100, 2 mM CaCl2 and EDTA-free Protease Inhibitor Cocktail (Roche, 11836170001)) were incubated with 10 ug Anti-T-cadherin (CDH13) (Sigma-Aldrich, ABT121) or IgG and 1.5 mg Dynabeads™ Protein G (Invitrogen, 10007D) using the indirect technique. Input and IP samples were separated by SDS-PAGE, transferred on nitrocellulose membranes (BioRad 170-4158) and probed with antibodies against Integrin β1 (1:5000 Abcam, ab183666) and CDH13 (1:1000 Sigma-Aldrich, ABT121). Proteins were then detected with Horseradish Peroxidase conjugated Goat anti-Mouse (1:50.000 Jackson ImmunoResearch Laboratories, 115-035-062) and Goat anti-Rabbit (1:50.000 Invitrogen, G21234). Proteins were revealed with Super Signal West Femto ECL (Thermo Scientific, 34095) and visualized with ChemiDoc Touch Imaging system (BioRad).

References from supplemental methods

1. Mandegar MA, Huebsch N, Frolov EB, Shin E, Truong A, Olvera MP *et al.* CRISPR Interference Efficiently Induces Specific and Reversible Gene Silencing in Human iPSCs. *Cell Stem Cell* 2016; **18**(4)**:** 541-553.

2. Frega M, Linda K, Keller JM, Gümüş-Akay G, Mossink B, van Rhijn J-R *et al.* Neuronal network dysfunction in a model for Kleefstra syndrome mediated by enhanced NMDAR signaling. *Nature Communications* 2019; **10**(1)**:** 4928.

3. Kondo T, Imamura K, Funayama M, Tsukita K, Miyake M, Ohta A *et al.* iPSC-based compound screening and in vitro trials identify a synergistic anti-amyloid β combination for Alzheimer's disease. *Cell reports* 2017; **21**(8)**:** 2304-2312.

4. Okita K, Matsumura Y, Sato Y, Okada A, Morizane A, Okamoto S *et al.* A more efficient method to generate integration-free human iPS cells. *Nature methods* 2011; **8**(5)**:** 409-412.

5. Takahashi K, Yamanaka S. Induction of pluripotent stem cells from mouse embryonic and adult fibroblast cultures by defined factors. *cell* 2006; **126**(4)**:** 663-676.

6. Mandegar MA, Huebsch N, Frolov EB, Shin E, Truong A, Olvera MP *et al.* CRISPR interference efficiently induces specific and reversible gene silencing in human iPSCs. *Cell stem cell* 2016; **18**(4)**:** 541-553.

7. Miyaoka Y, Chan AH, Judge LM, Yoo J, Huang M, Nguyen TD *et al.* Isolation of single-base genome-edited human iPS cells without antibiotic selection. *Nature methods* 2014; **11**(3)**:** 291-293.

8. Frega M, Linda K, Keller JM, Gümüş-Akay G, Mossink B, van Rhijn J-R *et al.* Neuronal network dysfunction in a model for Kleefstra syndrome mediated by enhanced NMDAR signaling. *Nature communications* 2019; **10**(1)**:** 1-15.

9. Vitale MR, Zöller JEM, Jansch C, Janz A, Edenhofer F, Klopocki E *et al.* Generation of induced pluripotent stem cell lines deficient for Cadherin 13 (UKWMPi002-A-1/B/C) Bassociated with neurodevelopmental disorders using CRISPR/Cas9. *Stem Cell Research* 2021**:** 102169.

10. Nadif Kasri N, Nakano-Kobayashi A, Malinow R, Li B, Van Aelst L. The Rho-linked mental retardation protein oligophrenin-1 controls synapse maturation and plasticity by stabilizing AMPA receptors. *Genes Dev* 2009; **23**(11)**:** 1289-1302.

11. Frega M, van Gestel SH, Linda K, van der Raadt J, Keller J, Van Rhijn JR *et al.* Rapid Neuronal Differentiation of Induced Pluripotent Stem Cells for Measuring Network Activity on Micro-electrode Arrays. *J Vis Exp* 2017; (119).

12. McCarthy KD, de Vellis J. Preparation of separate astroglial and oligodendroglial cell cultures from rat cerebral tissue. *J Cell Biol* 1980; **85**(3)**:** 890-902.

13. Livak KJ, Schmittgen TD. Analysis of relative gene expression data using real-time quantitative PCR and the 2(-Delta Delta C(T)) Method. *Methods* 2001; **25**(4)**:** 402-408.

14. Cao J, Packer JS, Ramani V, Cusanovich DA, Huynh C, Daza R *et al.* Comprehensive single-cell transcriptional profiling of a multicellular organism. *Science* 2017; **357**(6352)**:** 661-667.

15. Bolger AM, Lohse M, Usadel B. Trimmomatic: a flexible trimmer for Illumina sequence data. *Bioinformatics* 2014; **30**(15)**:** 2114-2120.

16. Dobin A, Davis CA, Schlesinger F, Drenkow J, Zaleski C, Jha S *et al.* STAR: ultrafast universal RNA-seq aligner. *Bioinformatics* 2013; **29**(1)**:** 15-21.

17. Smith T, Heger A, Sudbery I. UMI-tools: modeling sequencing errors in Unique Molecular Identifiers to improve quantification accuracy. *Genome Res* 2017; **27**(3)**:** 491-499.

18. Anders S, Pyl PT, Huber W. HTSeq--a Python framework to work with high-throughput sequencing data. *Bioinformatics* 2015; **31**(2)**:** 166-169.

Supplementary Figures

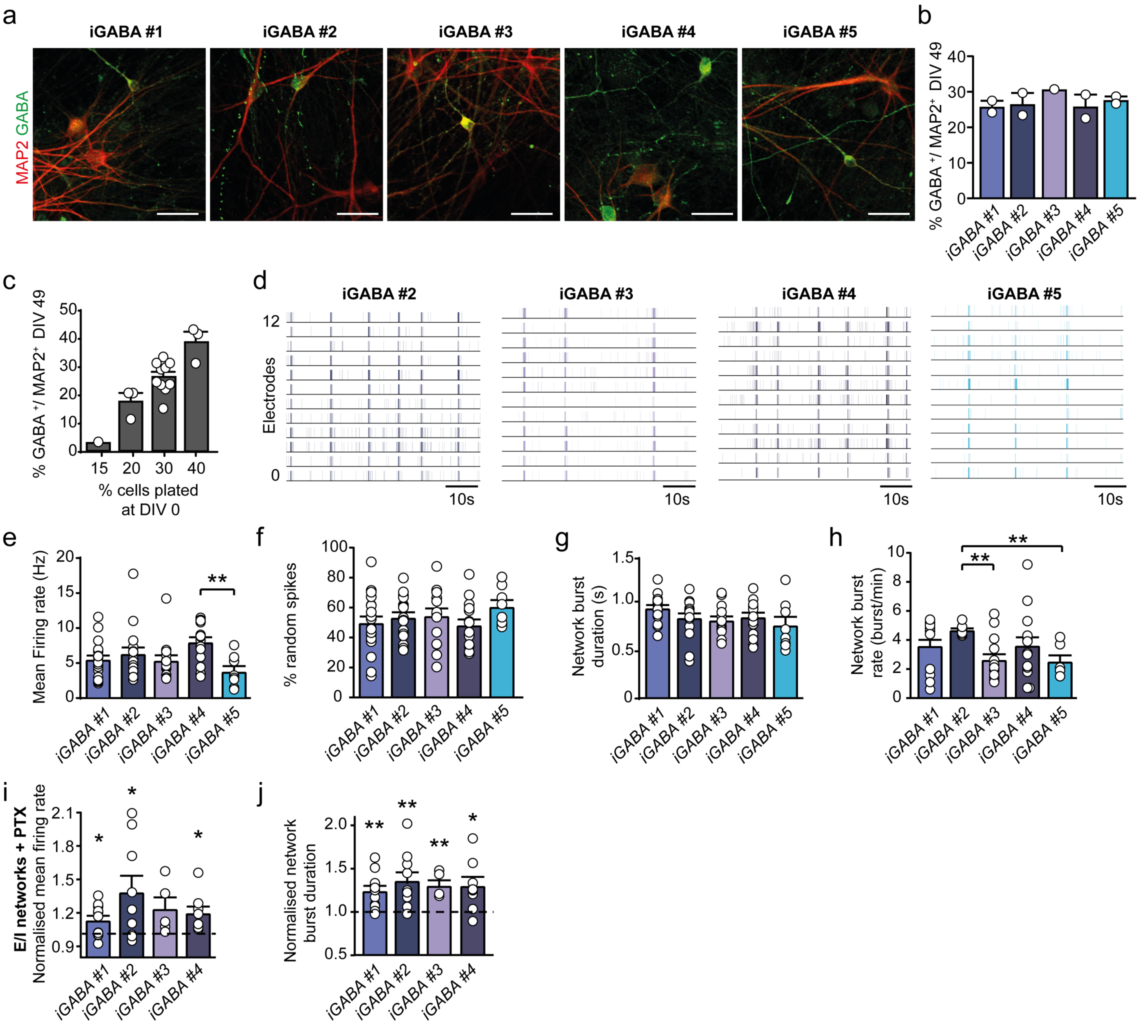

Supplementary figure 1. *Ascl1* overexpression robustly generates GABAergic neurons from several hiPSC lines that shape neuronal network activity. (**a-b**) Representative images (**a**) and quantification (**b**) of the percentage of MAP2 and GABA co-localized neurons in networks derived from iGABA#1-#5 in co-culture with iGLU#2 at an E:I ratio of 75:25 at DIV 49. (**c**) Quantification of the actual percentage of MAP2 and GABA co-localized neurons in E:I cultures at DIV 49 plotted against the theoretical percentage of GABAergic neurons plated at DIV 0. (**d**) Representative raster plot of 60 s of recording from E:I 75:25 cultures generated with iGABA#2-#5. (**e-h**) Quantification of neuronal network activity including (**e**) the mean firing rate (**f**) percentage of random spikes, (**g**) network burst duration and (**h**) network burst rate detected in E:I 75:25 cultures generated with iGABA#1-#5 at DIV 49 (sample size n for iGABA#1 n=19, iGABA#2 n=16, iGABA#3 n=14, iGABA#4 n=15 and iGABA#5

n=8 individual wells from 3 neuronal preparations. Kruskal Wallis ANOVA with post hoc Dunn's correction for multiple testing were performed between conditions). (**i-j**) Quantification of the changes in (**i**) mean firing rate and (**j**) network burst duration of E:I 75:25 cultures generated with iGABA#1-4 upon treatment with 100 μM Picrotoxin (PTX) at DIV 49. Dotted line represents the normalized condition before PTX treatment (iGABA#1 n=11, iGABA#2 n=9, iGABA#3 n=5, iGABA#4 n=8 individual wells from 3 neuronal preparations. Paired T-test was performed between network activity pre, and post treatment). DIV: Days in vitro. All data represent means ± SEM. * $p < 0.05$; ** $p < 0.01$; *** $p < 0.001$. Scale bar is 30 μM.

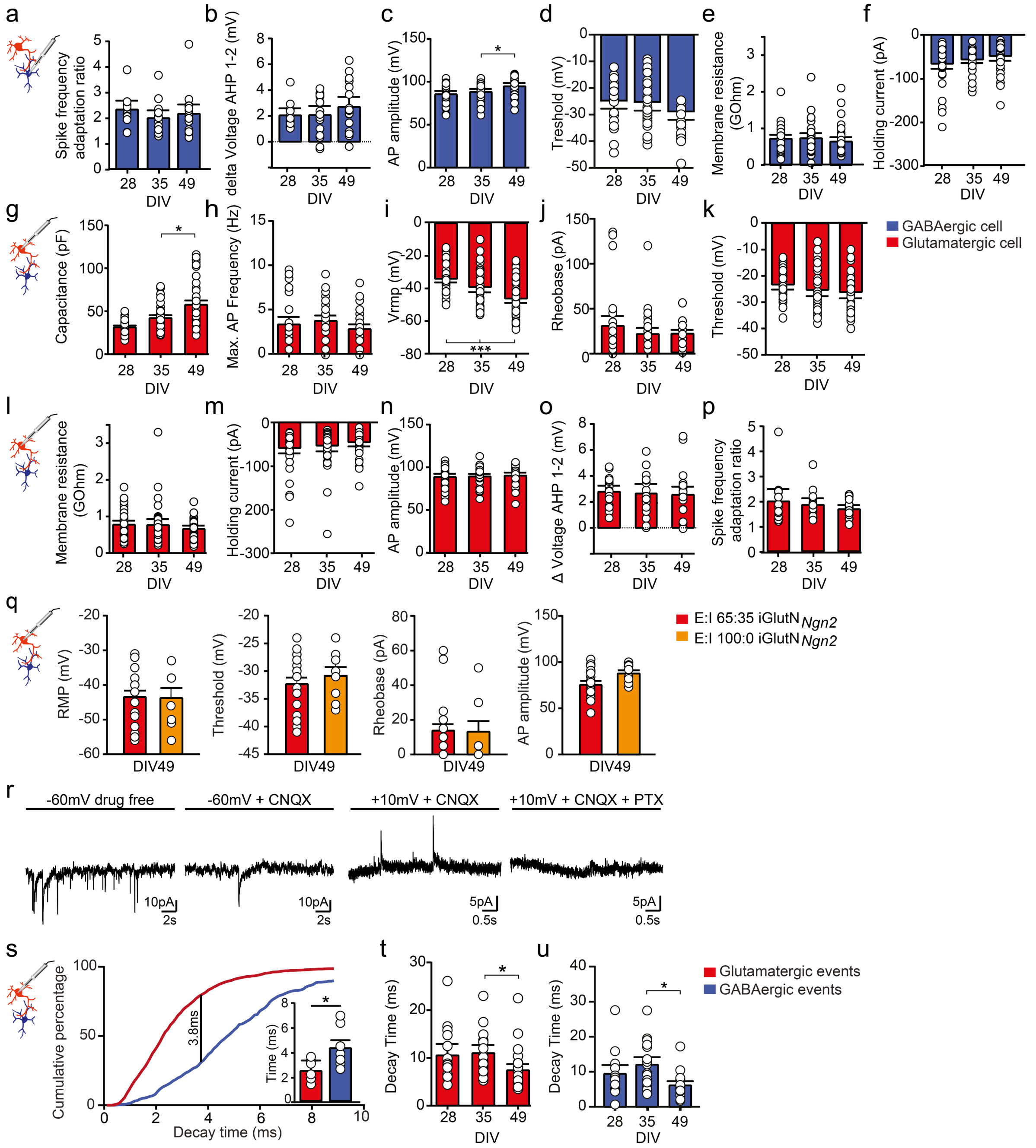

Supplementary figure 2. iGLU$_{Ngn2}$ and iGABA$_{A\text{-}FSK}$ mature over development. (**a-f**) Passive and active intrinsic properties recorded from iGABA$_{A\text{-}FSK}$ in an E:I 65:35 culture (Sample size for DIV 28 n=39, DIV 35 n=38, DIV 49 n=41 cells from 3 batches). (**g-p**) Passive and active intrinsic properties recorded from iGLU$_{Ngn2}$ in an E:I culture (Sample size for DIV 28 n=42, DIV 35 n=40, DIV 49 n=44 recorded cells from 3 individual neuronal preparations). (**q**) Intrinsic properties recorded from iGLU$_{Ngn2}$ in E:I 65:35 (red) versus iGLU$_{Ngn2}$ in an E:I 100:0 (yellow) only culture (iGLU$_{Ngn2}$ in E:I 65:35 networks

n=23 and $iGLU_{Ngn2}$ in E:I 100:0 networks n=8 recorded cells from 2 individual neuronal preparations). (**r**) Representative traces of spontaneous network activity (i.e. Glutamatergic and GABAergic sPSCs) under drug free conditions (panel 1), when AMPA receptors are blocked with 6-cyano-7-nitroquinoxaline-2,3-dione (CNQX, GABAergic sPSCs in panel 2 and 3), or when AMPA and GABA receptors are blocked with CNQX and Picrotoxin (PTX, panel 4) at DIV 49. (**s**) Cumulative plot of decay time of either GABAergic or glutamatergic events. Largest difference at 3.8 ms explains 78% percent of variance. (**s-u**) Average decay time calculated from spontaneous activity that was split at decay time of 3.8 ms to distinguish the (**t**) Glutamatergic and (**u**) GABAergic events at DIV 49. DIV: Days in vitro. All data represent means ± SEM. * $p < 0.05$, *** $p < 0.001$. Mann-Whitney test with post hoc Bonferroni correction for multiple testing was performed between DIVs.

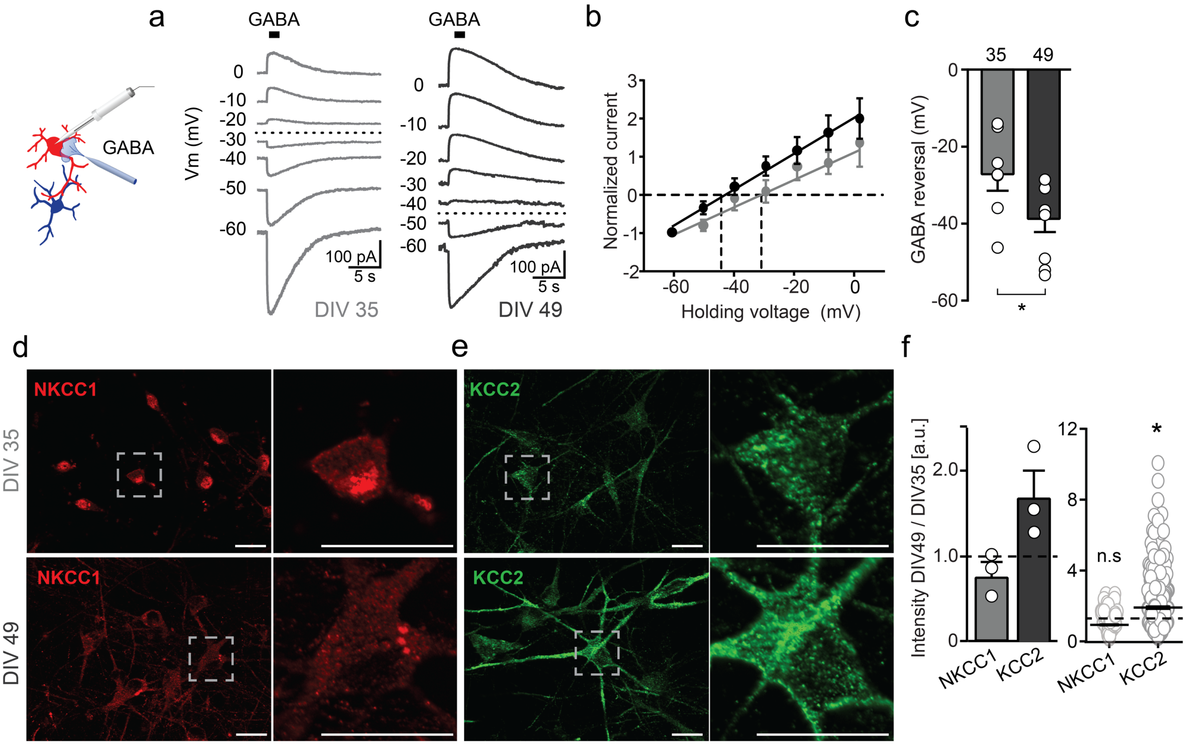

Supplementary figure 3. iGABA$_{A\text{-}FSK}$ become functionally inhibitory at DIV 49. (**a**) Representative traces showing GABA-evoked currents of iGLU$_{Ngn2}$ under various holding potentials at DIV 35 and -49. (**b**) Quantification of GABA-evoked responses. Dashed lines represent GABA reversal potential. (**c**) Quantified results of the reversal potential at DIV 35 and DIV 49 (DIV 35 n=7 and DIV 49 n=10 cells from 2 neuronal preparations. Mann-Whitney test with post hoc Bonferroni correction for multiple testing was performed between DIVs). (**d-e**) Representative (**d**) NKCC1 and (**e**) KCC2 immunostaining in E/I networks at DIV 35 and -49. (**f**) NKCC1 and KCC2 intensity measurements at DIV 49 normalized to the expression levels of DIV 35 (dashed line). Left panel: each data point represents one neuronal preparation. Right panel: Each data point represents the normalized intensity of one cell (NKCC1 DIV 35 n=206; NKCC1 DIV 49 n=153; KCC2 DIV 35 n=256; KCC2 DIV 49 n=237 cells analyzed from 3 different neuronal preparations. Nested One-Way ANOVA with post hoc Sidak correction for multiple testing was performed). DIV: Days in vitro. All data represent means ± SEM. * $p < 0.05$; *** $p < 0.001$. Scale bar 10 µM.

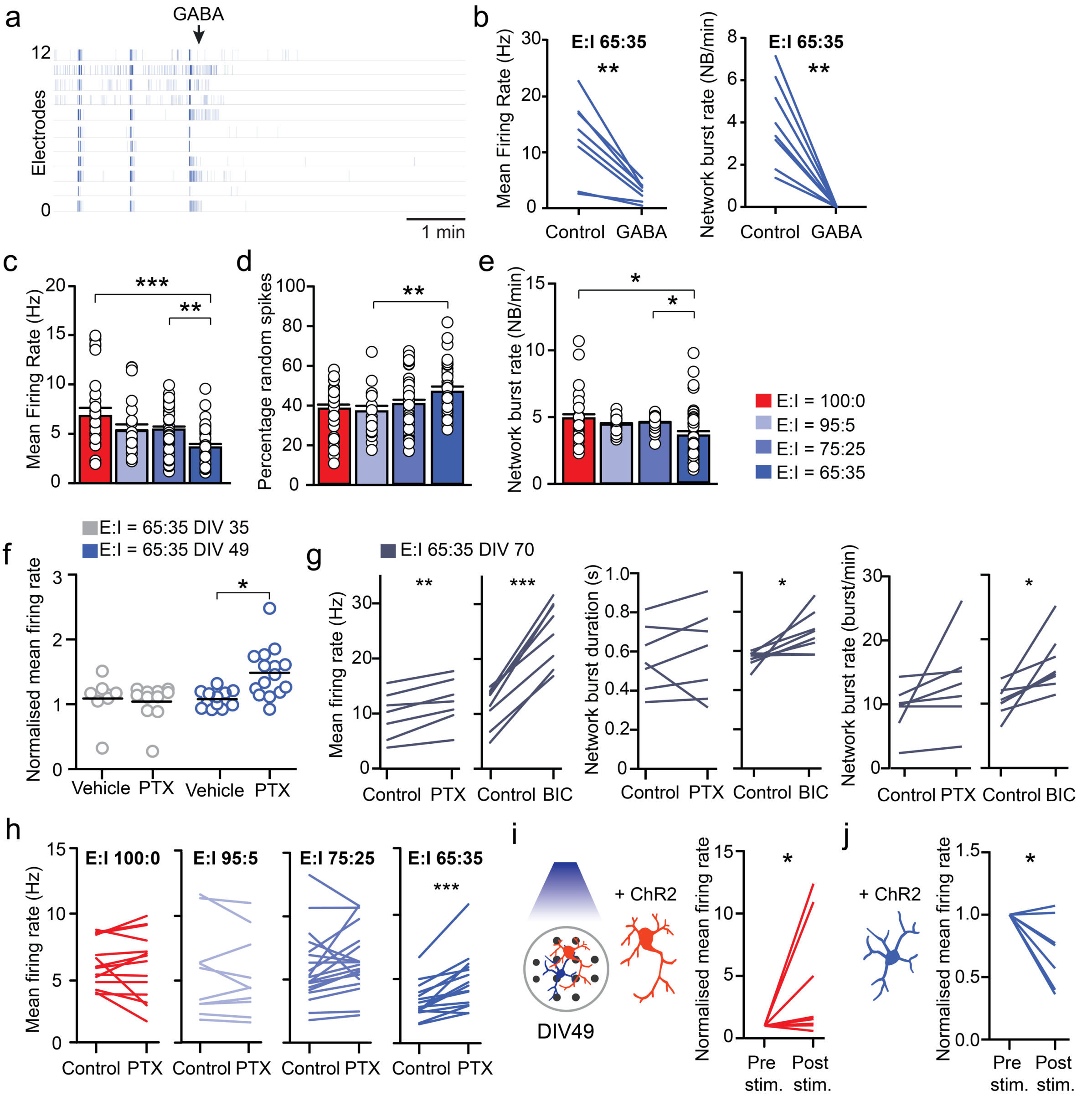

Supplementary figure 4. Functional GABAergic modulation in E/I networks is depending on the hyperpolarizing GABA shift and scalable to the percentage iGABA$_{\text{A-FSK}}$ present in the network. (**a**) Representative raster plot of an E:I 65:35 network treated with 100 µM Gamma-Aminobutyric acid (GABA) at DIV 49. (**b**) Paired analysis of the mean firing rate (MFR, left) and network burst rate (NBR, right) before and after treatment with GABA in E:I 65:35 networks at DIV 49 (Sample size n for E:I 65:35 networks n=9 individual wells, paired T-test was performed between pre and post conditions). (**c-e**) Quantifications of (**c**) MFR, (**d**) percentage of random spikes and (**e**) NBR in E:I networks at DIV 49 (E:I 100:0 n=29, E:I 95:5 n=20, E:I 75:25 n=38 and E:I 65:35 n=38 individual wells, Kruskal-Wallis Two-way ANOVA was performed and corrected for multiple testing using Dunn's). (**f**) MFR of vehicle

or 100 μM PTX treated E:I 65:35 networks, normalized to their respective baseline recording at DIV 49 (Sample size for DIV 35 + vehicle n= 8; DIV 35 + PTX n=11; DIV 49 + vehicle n=12 and DIV 49 + PTX n=15 individual wells, Mann-Whitney-test was performed, *p* values were corrected for multiple testing using Bonferroni method). (**g**) Paired analysis of the MFR, NBD and NBR in response to PTX or Bicuculline (BIC) treatment in E:I 65:35 networks at DIV 70 (Sample size for PTX treated cultures n=7 and BIC treated wells n=8 individual wells, paired T-test was performed between pre and post conditions). (**h**) Paired analysis of the MFR before and after treatment with 100 μM PTX at DIV 49 (100:0 n=15, 95:5 n=10, 75:25 n=19 and 65:35 n=15 individual wells, paired T-test was performed between pre and post conditions). (**i-j**) MFR of 65:35 networks upon optogenetic activation of $iGLU_{Ngn2}$ (**i**) or $iGABA_{A\text{-}FSK}$ (**j**) neurons respectively at DIV 49 (sample size n=7 individual wells for both conditions, paired T-test (Wilcoxon rank-sum) was performed between pre and post conditions). DIV: Days in vitro. All data represent means ± SEM. * $p < 0.05$; ** $p < 0.01$; *** $p < 0.001$.

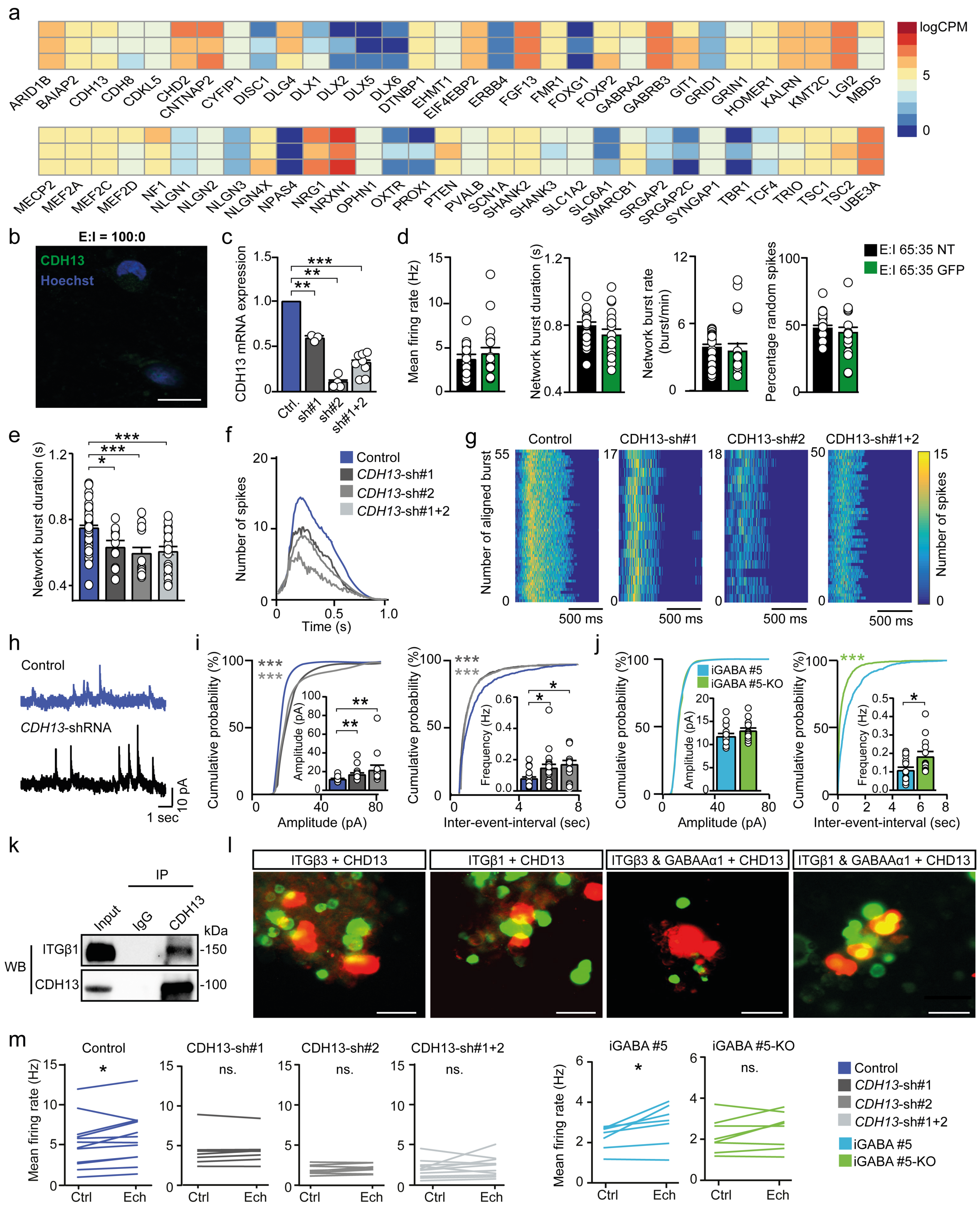

5upplementary figure 5. E/I cultures pose a valid model to study cell-type specific interactions of NDD genes to network dysfunction; an example for CDH13-deficiency. (**a**) Bulk RNA sequencing analysis of E:I 65:35 networks at DIV 49 (3 biological replicates). (**b**) CDH13 expression in iGLU$_{Ngn2}$ neurons at DIV 49. (**c**) *CDH13* mRNA expression in E:I 65:35 networks at DIV 49 following shRNA mediated knockdown (*CDH13*-sh#1 n=3, *CDH13*-sh#2 n=4, *CDH13*-sh#1+2 n=8 neuronal preparations

from which one sample is isolated for analysis. Paired T-test with was performed between the *PPIA* and *CDH13* mRNA expression). (**d**) Network activity of non-treated (NT) and GFP empty vector controls (GFP) controls at DIV 49 (Sample size n for E:I 65:35 NT n= 20 and E:I 65:35 GFP empty vector n= 20 individual wells from 3 neuronal preparations). (**e**) Quantification of the network burst duration of E/I 65:35 control and CDH13-deficient networks at DIV 49 (Control n=38, *CDH13*-sh#1 n=10 wells, *CDH13*-sh#2 n=12 wells, *CDH13*-sh#1+2 n=31 wells from 3 neuronal preparations. Kruskal Wallis ANOVA with post hoc Dunn's correction for multiple testing was performed between conditions). (**f**) Average network burst shape of representative cultures from E/I 65:35 control and CDH13-deficient networks at DIV 49 (Sample size for control n=26 individual wells, *CDH13*-sh#1 n=11 wells, p= 0.0129; *CDH13*-sh#2 n=8 wells, p<0.0001 and *CDH13*-sh#1+2 n=12 wells, p=0.00071). (**g**) Representative network burst alignment from E/I 65:35 control and CDH13-deficient networks at DIV 49. (**h**) Example trace of spontaneous inhibitory postsynaptic currents (sIPSC) activity from E/I 65:35 control and CDH13-deficient networks at DIV 70. (**i, j**) Cumulative distribution and quantification (inset) of sIPSC amplitude and frequency in E/I 65:35 control and CDH13-deficient networks (Control n=18, *CDH13*-sh#1 n=19 and *CDH13*-sh#2 n=13 recorded cells from 2 neuronal preparations, P<0.0001 for both frequency and amplitude; iGABA#5 n= 16 and iGABA#5-KO n= 13 cells from 2 neuronal preparations, frequency P<0.0001). Cumulative distributions were compared using Kolmogorov-Smirnov test with post hoc Bonferroni correction. Quantifications were compared using Kruskal Wallis ANOVA with post hoc Dunn's correction (shRNAs) or Mann-Whitney-test with Bonferroni correction (CRISPR) for multiple testing. (**k**) Western blot showing co-immunoprecipitation of CDH13 with ITGβ1 in E:I 50:50 networks at DIV 49. (**l**) Representative fluorescent images of cell aggregation assay (scale bar is 50 μM). (**m**) Quantification of the mean firing rate of pre- and post echistatin treated E:I 65:35 networks at DIV 49 (Sample size n for control n=16, *CDH13*-sh#1 n=7, *CDH13*-sh#2 n=7, *CDH13*-sh#1+2 n=8, iGABA#5 n= 7 and iGABA#5-KO n= 8 individual wells from 3 neuronal preparations. Paired T-test with was performed between pre and post echistatin treatment conditions). DIV: Days in vitro. NDD: Neurodevelopmental disorder. All data represent means ± SEM. * $p < 0.05$; ** $p < 0.01$; *** $p < 0.001$.

Supplementary tables

Supplementary table 1: Count table with raw counts/(log)cpm values displayed in **Figure 1f** and **Supplementary figure 5a**.

**Panel**		**DIV**	**Mean**	**SEM**	***p*-value**	
**Fig. 1h**	Rmp	28	-32.3	1.5		
		35	-38.3	1.6	<0.001	DIV 49
		49	-50.2	1.3	<0.001	DIV 28, DIV 35
**Fig. 1i**	Capacitance	28	37.6	1.7		
		35	43.1	2.2	<0.05	DIV 49
		49	57.8	3.5	<0.001	DIV 28
**Fig. 1l**	Correlated synaptic input	28	0.19	0.08		
		35	0.24	0.08	<0.001	DIV 49
		49	0.65	0.11	<0.001	DIV 28, DIV 35
**Sup. Fig. 2c**	Ap amplitude	28	85.5	2.0		
		35	88.1	1.8		
		49	94.6	2.1	<0.05	DIV 35
**Sup. Fig. 2g**	Capacitance	28	32.3	1.2		
		35	43.2	2.3		
		49	58.7	3.9	<0.001	DIV35
**Sup. Fig. 2i**	Rmp	28	-34.0	1.3		
		35	-39.1	1.8	<0.001	DIV28, DIV49
		49	-46.2	1.5	<0.001	DIV28, DIV35
**Sup. Fig. 2s**	Decay time	49	2.39	0.32		
		49	4.23	0.65	<0.05	
**Sup. Fig. 2t**	Decay time	28	10.6	1.5		
		35	11.0	1.1		
		49	7.4	7.4	<0.05	DIV35
**Sup. Fig. 2u**	Decay time	28	9.45	2.35		
		35	12.05	2.01		
		49	6.12	0.90	<0.05	DIV35

Supplementary table 2. Statistics of intrinsic properties from E/I networks in **Figure 1** and **Supplementary figure 2**. **Figure 1h, i and Sup. Fig. 2c, u**: Sample size for intrinsic properties of $iGABA_{A\text{-}FSK}$ at DIV 28 n=39, DIV 35 n=38, DIV 49 n=41 recorded cells from 3 neuronal preparations. **Figure 1l:** Sample size of correlated synaptic inputs at DIV 28 n=55, DIV 35 n=38, DIV 49 n=42 recorded cells from 3 neuronal preparations. **Sup. Fig. 2g, i, t:** Sample size for DIV 28 n=42, DIV 35 n=40, DIV 49 n=44 cells from 3 batches. All data represent means ± SEM. Two-way ANOVA with Tukey correction for multiple testing was used to compare between DIVs. DIV= Days in vitro.

		**Cells**	**Batches**	**Mean**	**SEM**	***p*-value**
**c**	GABA reversal DIV 35	7	2	-27.14	4.29	0.0136
	GABA reversal DIV 49	10	2	-39.33	2.86	
**f**	NKCC1 DIV 49/DIV 35	294	3	0.93	0.03	n.s
	KCC2 DIV 49/DIV 35	450	3	1.91	0.07	0.0264

Supplementary table 3. Statistics of **Supplementary figure 3c, f**. All data represent means ± SEM. * p < 0.05; ** p < 0.01; *** p < 0.001. Batch number indicates the amount of neuronal preparations. Mann-Whitney test with post hoc Bonferroni correction for multiple testing was performed between DIVs in panel c. Nested One-Way ANOVA with post hoc Sidak correction for multiple testing was performed on normalized NKCC1 and KCC2 data in panel f. DIV= Days in vitro.

		E:I=100:0			E:I=65:35				
	DIV	Mean	SEM	n	Mean	SEM	n	*p*-value E:I 65:35 vs 100:0	*p*-value development
**NBD (MS)**	35	1228.60	81.38	25	1059.57	41.74	36	0.2026	
	42	1108.37	57.01	30	853.13	24.38	39	0.0006	
	49	1114.40	31.42	29	750.19	18.46	37	<0.0001	<0.0001
**NBR (B/MIN)**	35	3.27	0.24	25	2.40	0.16	40	0.0143	
	42	4.45	0.27	30	2.62	0.21	39	<0.0001	
	49	4.90	0.33	29	3.562	0.32	37	0.0150	<0.0001
**MFR (HZ)**	35	3.62	0.54	25	2.322	0.27	40	0.1053	
	42	6.43	0.85	30	2.98	0.28	39	0.0014	
	49	7.06	0.67	29	3.68	0.31	38	<0.0001	<0.0001
**PRS (%)**	35	53.37	3.86	25	55.87	2.83	40	0.9382	
	42	44.26	3.06	30	53.33	2.38	39	0.0664	
	49	37.54	2.43	29	47.52	2.18	38	0.0100	<0.0001

Supplementary table 4. Statistics of **Figure 2g-j**. All data represent means ± SEM. * p < 0.05; ** p < 0.01; *** p < 0.001. Mixed model Two-way ANOVA was performed between DIVs, p values were corrected for multiple comparisons using Sidak's. NBD= Network burst duration, NBR= Network burst rate, MFR= Mean firing rate, PRS= percentage of random spikes, n= number of wells, DIV= Days in vitro.

**Panel**		**Basal condition**			**Post PTX**			***p*-value**
		**Mean**	**SEM**	**N**	**Mean**	**SEM**	**N**	
**e**	iGABA#1	5.270	0.581	19				iGABA#4 vs iGABA#5 = 0.00697
	iGABA#2	6.070	0.909	16				
	iGABA#3	5.127	0.758	14				
	iGABA#4	7.710	0.695	15				
	iGABA#5	3.596	0.774	8				
**h**	iGABA#1	3.794	0.390	17				iGABA#2 vs iGABA#3= 0.00612
	iGABA#2	4.708	0.090	14				iGABA#2 vs iGABA#5= 0.00534
	iGABA#3	2.629	0.392	14				
	iGABA#4	3.613	0.578	15				
	iGABA#5	2.343	0.428	7				
**i**	iGABA#1	1	-	11	1.132	0.043	11	0.0115
	iGABA#2	1	-	9	1.385	0.148	9	0.0315
	iGABA#3			5	1.235	0.104	5	0.0853
	iGABA#4	1	-	8	1.197	0.060	8	0.0134
**j**	iGABA#1	1	-	11	1.24	0.062	11	0.0030
	iGABA#2	1	-	9	1.359	0.099	9	0.0054
	iGABA#3	1	-	5	1.301	0.065	5	0.0096
	iGABA#4	1	-	8	1.3	0.105	8	0.0248

Supplementary table 5. Statistics of **Supplementary figure 1e, h-j**. All data represent means ± SEM. * p < 0.05; ** p < 0.01; *** p < 0.001. N represents the number of recorded MEAs from 2 neuronal preparations. Kruskal Wallis ANOVA with post hoc Dunn's correction for multiple testing was performed between Ascl1 lines in panel e, h. Other comparisons were non-significant. Paired T-test or Wilcoxon matched-pairs signed rank test was performed between network activity pre, and post treatment in panel i, j. Basal = before treatment/no treatment, PTX= Picrotoxin.

	E:I=100:0			E:I=95:5			E:I=75:25			E:I=65:35			
	**M**	**SEM**	**n**	**M**	**SEM**	**n**	**M**	**SEM**	**n**	**M**	**SEM**	**n**	***p*-value mult. comp.**
**NBD (MS)**	1114	31.42	30	1045	55.98	19	901.8	26.91	42	751.2	17.53	39	100:0 / 75:25 = 0.0006<br>100:0 / 65:35 = <0.0001<br>95:5 / 65:35 = 0.0002<br>75:25 / 65:35 = 0.0015
**NBR (B/MIN)**	4.90	0.34	30	4.52	0.14	19	4.57	0.07	42	3.62	0.33	39	100:0 / 65:35 = 0.0220<br>75:25 / 65:35 = 0.0209
**MFR (HZ)**	7.06	0.67	30	5.42	0.59	19	5.33	0.37	42	3.68	0.31	39	100:0 / 65:35 = <0.001<br>75:25 / 65:35 = 0.0097
**PRS (%)**	37.54	2.43	30	34.31	2.71	19	40.62	2.31	42	48.55	2.11	39	95:5 / 65:35 = 0.0015

Supplementary table 6. Statistics of **Figure 3d** and **supplementary figure 4c-e**. All data represent means ± SEM. * p < 0.05; ** p < 0.01; *** p < 0.001. Kruskal Wallis one-way ANOVA was performed between ratio's; p values were corrected for multiple comparisons using Dunn's. Multiple comparison statistics are mentioned last column, compared ratios were split by '/'. Other comparisons were non-significant. M=Mean, n= number of wells, NBD= Network burst duration, NBR= Network burst rate, MFR= Mean firing rate, PRS= percentage of random spikes.

			Basal		Post PTX/BIC			
			**Mean**	**SEM**	**Mean**	**SEM**	**n**	***p*-value**
**Fig. 3g**	NBD (ms)	DIV 49	1.01	0.05	1.33	0.08	15	0.0017
**Fig. 3j**	NBD (ms)	75:25	897.2	26.21	1108	37.94	18	<0.0001
**Fig. 3k**	NBD (ms)	65:35	727.2	41.23	967.7	76.26	15	0.001
**Sup. Fig. 4b**	MFR (Hz)	DIV49	12.32	2.48	2.81	0.58	8	0.0021
	NBR (b/min)	DIV49	4.05	0.72	0	0	8	0.0078
**Sup. Fig. 4f**	MFR (Hz)	DIV 49	1.12	0.04	1.49	0.10	15	0.0044
**Sup. Fig. 4g**	MFR (Hz)	DIV 49 + PTX	9.684	1.602	12.16	1.583	7	0.0018
		DIV 49 + BIC	11.25	1.310	24.87	2.040	8	<0.0001
	NBD (ms)	DIV 49 + BIC	0.560	0.014	0.696	0.037	8	0.0220
	NBR (b/min)	DIV 49 + BIC	10.44	0.789	16.28	1.536	8	0.0105
**Sup. Fig. 4h**	MFR (Hz)	65:35	3.28	1.37	4.78	2.18	15	0.0003
**Sup. Fig. 4i**	Norm. MFR	ChR2 in iGLU	1	-	3.87	1.66	8	0.0391
**Sup. Fig. 4j**	Norm. MFR	ChR2 in iGABA	1	-	0.81	0.13	7	0.0319

Supplementary table 7. Statistics of **Figure 3g, j and k** and **Supplementary figure 4b, f-j**. All data represent means ± SEM. * p < 0.05; ** p < 0.01; *** p < 0.001. Paired T-test or Wilcoxon matched-pairs signed rank test was performed between network activity pre, and post treatment in**.** Basal = before treatment, PTX= Picrotoxin, BIC= Bicuculline, NBR= Network burst rate, NBD= Network burst duration, MFR= Mean firing rate, n= number of wells, DIV= Days in vitro.

	Control		*CDH13* mRNA expression			
	**Mean**	**SEM**	**Mean**	**SEM**	**n**	***p*-value**
***CDH13*-shRNA#1**	1	-	0.6027	0.0248	3	0.0039
***CDH13*-shRNA#2**	1	-	0.119	0.078	4	0.032
***CDH13*-shRNA#1+2**	1	-	0.323	0.042	8	0.0007

Supplementary table 8. Statistics of **supplementary figure 5c**. All data represent means ± SEM. Mann-Whitney test was performed between control and *CDH13*-shRNA#1+2 transduced networks. Significance was corrected for multiple comparisons using Bonferroni. Paired T-test was performed between *CDH13*-shRNA#1, *CDH13*-shRNA#2 and controls. N represents the number neuronal preparations from which one sample is isolated for analysis.

Gene	Forward (5'->3')	Reverse (5'->3')
PPIA	AAGTGACAAGGGCTTTCGTG	AAGCCCTCATTGGCAGTTAC
CDH13	TAAGGGAAACGACAAGCACGC	CTCAGAGCAACTAAGCCGCC
CDH13*-shRNA *#1	CCGGGCTTTCTGTTGTCAAACCATTCTCGAGAATGGTTTGACAACAGAAAGCTTTTTG	
CDH13-shRNA #2	CCGGGCAGAAAGTGTTCCATATCAACTCGAGTTGATATGGAACACTTTCTGCTTTTT	

Supplementary table 9. Forward and reverse primer sequences and *CDH13* targeting short hairpin RNA sequences.

Panel			DIV	Mean	SEM	N	*p*-value
**Fig. 4d**	VGAT intensity (a.u.)	Control	49	31881	1378	25	<0.001
		CDH13-sh#1+2	49	50029	1570	26	
		iGABA#5	49	30912	4183	13	0.0253
		iGABA#5-KO	49	46182	4939	15	
**Fig. 4f, j and Sup. fig. 5e**	NBD (ms)	Control	49	0.751	0.018	49	
		CDH13-sh#1	49	0.639	0.036	10	Control vs *CDH13*-sh#1 = 0.0378
		CDH13-sh#2	49	0.590	0.042	12	Control vs *CDH13*-sh#2 = 0.0008
		CDH13-sh#1+2	49	0.613	0.025	31	Control vs *CDH13*-sh#1+2 = 0.0002
		iGABA#5	49	0.517	0.018	20	0.0074
		iGABA#5-KO	49	0.425	0.023	21	
	NBR (burst/min)	Control	49	3.650	0.284	49	Control vs *CDH13*-sh#1 = 0.0327
		CDH13-sh#1	49	1.936	0.332	10	Control vs *CDH13*-sh#2 = 0.0022
		CDH13-sh#2	49	1.550	0.340	12	Control vs *CDH13*-sh#1+2 = 0.032
		CDH13-sh#1+2	49	2.534	0.364	31	
		iGABA#5	49	4.405	0.3288	20	0,0584
		iGABA#5-KO	49	3.61	0.3544	21	
	PRS (%)	Control	49	47.27	2.32	49	Control vs *CDH13*-sh#2 = 0.0266
		CDH13-sh#1	49	53.32	9.158	10	Control vs *CDH13*-sh#1+2 <0.001
		CDH13-sh#2	49	61.76	5.355	12	
		CDH13-sh#1+2	49	67.74	2.69	31	
		iGABA#5	49	39.43	2.795	20	<0.001
		iGABA#5-KO	49	60.34	2.834	21	
	MFR (Hz)	Control	49	3.749	0.28	49	Control vs *CDH13*-sh#2 = 0.0028
		CDH13-sh#1	49	3.410	0.386	10	Control vs *CDH13*-sh#1+2 = 0.0005
		CDH13-sh#2	49	1.864	0.232	12	

		CDH13-sh#1+2	49	2.283	0.261	31	
		iGABA#5	49	2.55	0.176	20	0.1491
		iGABA#5-KO	49	2.209	0.163	21	
**Sup. Fig. 5i**	sIPSC Amplitude	Control	70	90.94	2.414	25	P<0.05
		CDH13-sh#1	70	85.00	2.763	19	0.00200
		CDH13-sh#2	70	84.20	2.613	13	0.00436
		iGABA#5	70	11.83	0.564	16	
		iGABA#5-KO	70	13.22	0.602	13	0.0916
**Sup. Fig. 5j**	sIPSC Frequency	Control	70	85.16	0.444	25	
		CDH13-sh#1	70	91.28	0.382	19	0.0429
		CDH13-sh#2	70	92.02	0.341	13	0.0102
		iGABA#5	70	0.104	0.0151	16	
		iGABA#5-KO	70	0.184	0.0268	13	0.0151

Supplementary table 10. Statistics from **Figure 4d, f, j and Supplementary figure 5e, i, j**. All data represent means ± SEM. * p < 0.05; ** p < 0.01; *** p < 0.001. Total VGAT intensity, MEA parameters and sIPSCs from iGABA#5 vs iGABA#5-KO were compared using Mann-Whitney ranked sum test with post hoc Bonferroni correction. Ordinary one-way ANOVA with Dunnett correction for multiple testing or Kruskal Wallis ANOVA with Dunn's correction for multiple testing was used to compare between *CDH13*-shRNA transduced wells and controls. NBD= Network burst duration, MFR= Mean firing rate, PRS= percentage of random spikes, NBR= Network Burst Rate, n= number of wells, DIV= Days in vitro.

**Panel**		**T60/T0**	**Compared to**	**T60/T0**	***p*-values**
**i**	mCherry	0.94 ± 0.01	CHD2	0.58 ± 0.01	0.0022
**i**	mCherry	0.94 ± 0.01	CDH13	0.68 ± 0.01	0.0004
**i**	CDH2	0.58 ± 0.01	CDH13	0.68 ± 0.01	0.0001
**k**	CDH13	0.68 ± 0.01	GABAAα1/β3	0.79 ± 0.04	0.0202
**k**	CDH13	0.68 ± 0.01	GABAAα1/β3+ CDH13	0.78 ± 0.06	0.0253
**l**	CDH13	0.68 ± 0.01	ITGβ1	0.87 ± 0.05	0.0256
**l**	CDH13	0.68 ± 0.01	ITGβ1+ CDH13	0.62 ± 0.02	0.0350
**l**	ITGβ1	0.87 ± 0.05	ITGβ1+ CDH13	0.62 ± 0.02	0.0476
**m**	CDH13	0.68 ± 0.01	ITGβ3	0.90 ± 0.03	<0.0001
**m**	CDH13	0.68 ± 0.01	ITGβ3+ CDH13	0.60 ± 0.04	0.0298
**m**	ITGβ3	0.90 ± 0.03	ITGβ3+ CDH13	0.60 ± 0.04	0.0008
**o**	ITGβ3	0.90 ± 0.03	GABAAα1/β3 + ITGβ3	0.82 ± 0.03	0.0466
**p**	CDH13+ ITGβ1/ GABAAα1/β3	0.63 ± 0.004	mCherry	0.94 ± 0.01	0.0238
**p**	CDH13+ ITGβ1/ GABAAα1/β3	0.63 ± 0.004	CDH13+ITGβ3/ GABAAα1/β3	0.93 ± 0.06	0.0065

Supplementary table 11. Statistics of **Figure 5i-p.** All data represent means ± SEM. * p < 0.05; ** p < 0.01; *** p < 0.001. Depending on normal distribution, either an unpaired T-test or Mann Whitney-U test is performed between conditions. DIV= Days in vitro.

Panel			Basal		Post echistatin			
			Mean	SEM	Mean	SEM	n	*p*-value
**Fig. 5r**	NBD (ms)	Control	0.78	0.040	0.97	0.052	16	0.0005
		CDH13-sh#1	0.71	0.026	0.69	0.041	7	0.3690
		CDH13-sh#2	0.65	0.065	0.71	0.078	7	0.3578
		CDH13-sh#1+2	0.69	0.066	0.62	0.080	8	0.1101
		iGABA#5	0.50	0.014	0.64	0.057	7	0.0383
		iGABA#5-KO	0.41	0.044	0.43	0.040	8	0.3553
**Sup. Fig. 5m**	MFR (Hz)	Control	5.00	0.70	5.49	0.75	17	0.0456
		CDH13-sh#1	4.25	0.79	4.33	0.70	7	0.5211
		CDH13-sh#2	1.87	0.21	2.02	0.19	8	0.1256
		CDH13-sh#1+2	1.94	0.34	2.67	0.38	11	0.3692
		iGABA#5	2.25	0.23	2.92	0.39	7	0.0294
		iGABA#5-KO	2.25	0.30	2.54	0.31	8	0.1597

Supplementary table 12. Statistics of **Figure 5r and Supplementary figure 5m.** All data represent means ± SEM. * $p < 0.05$; ** $p < 0.01$; *** $p < 0.001$. Paired T-test was performed between pre and post-treatment conditions at DIV 49. Basal = before treatment, DIV= Days in vitro.

Primary antibody	Dilution, manufacturer
**Mouse anti-MAP2**	1:1000, Sigma M4403
**Guinea pig anti-MAP2**	1:1000, Synaptic Systems 188004
**Mouse anti-Calbindin**	1:500, Swant 300
**Mouse anti-Calretinin**	1:500, Swant 6B3
**Mouse anti-GAD67**	1:400, Millipore MAB5406
**Rabbit anti-GAD67**	1:500, Synaptic systems 198 013
**Rabbit anti-GABA**	1:1000, Sigma A2052
**Goat anti-Somatostatin**	1:500, Santa Cruz sc-7819
**Rabbit anti-parvalbumin**	1:500, Swant PV27
**Mouse anti-parvalbumin**	1:1000, Sigma P3088
**Mouse anti-Gephyrin**	1:500, Synaptic Systems 147011
**Rabbit anti-VGAT**	1:500, Synaptic systems 131 013
**Guinea pig anti-VGAT**	1:50, Synaptic systems 131 004
**Mouse anti-human Nuclei**	1:100, Millipore MAB1281
**Rabbit anti-Cadherin 13**	1:500, Millipore ABT121
**Rabbit anti-Cadherin 13**	1:1000, Sigma-Aldrich ABT121
**Mouse anti- Integrin β1**	1:5000, Abcam, ab183666
**Mouse anti- Integrin β1**	1:250, Abcam ab24693
**Mouse anti- Integrin β3**	1:100 Merck MAB1976
**Rabbit anti-NKCC1**	1:500, Abcam ab59791
**Rabbit anti-KCC2**	1:500, Abcam ab49917
**Mouse anti-MEF2C**	1:100, Novus Biologicals NBP2-00493
**Rabbit anti-SYT2**	1:250, Synaptic systems 105 123

Secondary antibody	Dilution, manufacturer
**Goat anti-guinea pig Alexa Fluor 647**	1:1000, Invitrogen A-21450
**Goat anti-guinea pig Alexa Fluor 568**	1:1000, Invitrogen A-11075
**Goat anti-rabbit Alexa Fluor 488**	1:1000, Invitrogen A-11034
**Goat anti-rabbit Alexa Fluor 568**	1:2000, Invitrogen A-11036
**Goat anti-rabbit Alexa Fluor 647**	1:1000, Invitrogen A-21245
**Goat anti-mouse Alexa Fluor 488**	1:1000, Invitrogen A-11029
**Goat anti-mouse Alexa Fluor 568**	1:2000, Invitrogen A-11031
**Goat anti-mouse Alexa Fluor 647**	1:1000, Invitrogen A-21236

**Donkey anti-goat Alexa Fluor 488**	1:1000, Invitrogen A-11055
**Donkey anti-goat Alexa Fluor 647**	1:1000, Invitrogen A-21447
**Donkey anti-goat Alexa Fluor 568**	1:1000, Invitrogen A-11057
**Donkey anti-rabbit Alexa Fluor 488**	1:1000, Invitrogen A-21206
**Donkey anti-rabbit Alexa Fluor 568**	1:1000, Invitrogen A-10042
**Goat anti-Rabbit HRP**	1:50.000 Invitrogen, G21234
**Goat anti-Mouse HRP**	1:50.000 Jackson ImmunoResearch Laboratories, 115-035-062

Supplementary table 13. Used primary and secondary antibodies
